# Neuroprotective Potentials of Marine Algae and Their Bioactive Metabolites: Pharmacological Insights and Therapeutic Advances

**DOI:** 10.3390/md18070347

**Published:** 2020-07-01

**Authors:** Md. Abdul Hannan, Raju Dash, Md. Nazmul Haque, Md. Mohibbullah, Abdullah Al Mamun Sohag, Md. Ataur Rahman, Md Jamal Uddin, Mahboob Alam, Il Soo Moon

**Affiliations:** 1 Department of Anatomy, Dongguk University College of Medicine, Gyeongju 38066, Korea; hannanbau@gmail.com (M.A.H.); rajudash.bgctub@gmail.com (R.D.); mahboobchem@gmail.com (M.A.); 2 Department of Biochemistry and Molecular Biology, Bangladesh Agricultural University, Mymensingh 2202, Bangladesh; sohag2010bmb.sust@gmail.com; 3 Department of Fisheries Biology and Genetics, Patuakhali Science and Technology University, Patuakhali 8602, Bangladesh; habib.332@gmail.com; 4 Department of Fishing and Post Harvest Technology, Sher-e-Bangla Agricultural University, Sher-e-Bangla Nagar, Dhaka 1207, Bangladesh; mmohib08@gmail.com; 5 Center for Neuroscience, Korea Institute of Science and Technology (KIST), Seoul 02792, Korea; ataur1981rahman@hotmail.com; 6 Graduate School of Pharmaceutical Sciences, College of Pharmacy, Ewha Womans University, Seoul 03760, Korea; hasan800920@gmail.com; 7 ABEx Bio-Research Center, East Azampur, Dhaka 1230, Bangladesh; 8 Division of Chemistry and Biotechnology, Dongguk University, Gyeongju 780-714, Korea

**Keywords:** seaweed, secondary metabolites, neuroprotection, Alzheimer’s disease, Parkinson’s disease, ischemic stroke, computer-aided drug discovery

## Abstract

Beyond their significant contribution to the dietary and industrial supplies, marine algae are considered to be a potential source of some unique metabolites with diverse health benefits. The pharmacological properties, such as antioxidant, anti-inflammatory, cholesterol homeostasis, protein clearance and anti-amyloidogenic potentials of algal metabolites endorse their protective efficacy against oxidative stress, neuroinflammation, mitochondrial dysfunction, and impaired proteostasis which are known to be implicated in the pathophysiology of neurodegenerative disorders and the associated complications after cerebral ischemia and brain injuries. As was evident in various preclinical studies, algal compounds conferred neuroprotection against a wide range of neurotoxic stressors, such as oxygen/glucose deprivation, hydrogen peroxide, glutamate, amyloid β, or 1-methyl-4-phenylpyridinium (MPP^+^) and, therefore, hold therapeutic promise for brain disorders. While a significant number of algal compounds with promising neuroprotective capacity have been identified over the last decades, a few of them have had access to clinical trials. However, the recent approval of an algal oligosaccharide, sodium oligomannate, for the treatment of Alzheimer’s disease enlightened the future of marine algae-based drug discovery. In this review, we briefly outline the pathophysiology of neurodegenerative diseases and brain injuries for identifying the targets of pharmacological intervention, and then review the literature on the neuroprotective potentials of algal compounds along with the underlying pharmacological mechanism, and present an appraisal on the recent therapeutic advances. We also propose a rational strategy to facilitate algal metabolites-based drug development.

## 1. Introduction

Neurons and supporting cells of the brain encounter degenerative changes during physiological or pathological aging, ischemic stroke, or other brain injuries [1]. The degenerative brain disorders such as Alzheimer’s disease (AD) and Parkinson’s diseases (PD) are the consequence of pathological brain aging, which are characterized by the region-specific loss of neurons [2]. Globally, these diseases account for the major causes of dementia among the elderly [3]. Although the exact etiologies of these brain disorders are not revealed yet, they share some common pathophysiology, such as oxidative stress (OS), neuroinflammation, mitochondrial dysfunction, protein misfolding, and defective protein clearance system that, in turn, make these diseases complicated [4,5], whereas, ischemic, traumatic, and other brain injuries, if not fatal, ensue secondary damage and constitute the appreciable causes of cognitive deficits among patients. Like neurodegenerative disorders, brain injuries also follow the same pathophysiology [6,7]. Whatever the forms of dementia disorder, the current therapeutic option can only alleviate symptoms, rather than halting the disease progression. Moreover, current drugs are associated with multiple side effects. Considering the tremendous social and economic impact of these diseases, scientists are, therefore, paying research efforts to discover the potential therapeutic agents that can target disease pathogenesis without causing undesirable effects in patient’s health. Although synthetic drugs have some advantages such as easy to develop, naturally-derived compounds have received priority as they are relatively well-tolerated. Natural compounds have been claimed to show anti-inflammatory, antioxidant, and immunomodulatory effects [8]. Compounds showing multiple pharmacological effects offer a better solution for the remedy of neurological disorders with complex pathomechanisms [9]. In the published literature, a significant quantity of natural products has been reported to show neuroprotective activity against a wide range of toxic insults [10,11]. Some of them have shown therapeutic promise in preclinical studies [12] and clinical trials [13,14].

Macroalgae, also known as seaweed, are among the highly abundant marine lives and potentially contribute to the renewable resources for food and industrial products [15,16,17]. Beyond this importance, algal metabolites, such as phenolics, alkaloids, terpenoids, carotenoids, phytosterols, and polysaccharides have attracted much attention to medicinal chemistry due to their structural uniqueness and functional diversity [17,18,19,20]. These biofunctional compounds have shown to provide neuroprotection in preclinical models of neurodegenerative diseases, ischemic stroke, brain trauma, diabetes, and obesity, among many others, owing to their antioxidant, anti-inflammatory, and immunomodulatory capacities [21,22,23,24,25,26,27,28]. Evidence suggests that algal metabolites, particularly fucoxanthin, fucosterol, and fucoidan could be potential leads for the development of therapy against CNS diseases [22,29,30,31]. Although the algal metabolite-based drug discovery progresses very slowly, the discovery of sodium oligomannate and its conditional approval as an anti-AD drug [32] raises hope for the future development of potential therapeutic agents from marine algae.

Over the last decade, some excellent works reviewed the neuroprotective effects of marine algae and their metabolites [21,22,23,29,33,34,35]. However, some of these reviews limited their scope either to a single pathogenic mechanism such as neuroinflammation [22] or to categorical brain disorders such as AD or PD [22,23,29,34,35]. Others have reviewed literature published a decade or half a decade ago [23,36]. Moreover, a few of them included reports that cover ischemic or other brain injuries. In the meantime, information on some potential algal compounds with neuroprotective activity has appeared in the scientific platform and there has also been significant progress in the clinical aspect. Addressing the knowledge gap and the possible limitations, offering a comprehensive review updating information on the neuroprotective effects of algal compounds and their therapeutic advances is timely. In this comprehensive review, we first briefly outline the pathobiology of neurodegenerative disorders, ischemic stroke, and traumatic brain injury and then provide pharmacological insights into the neuroprotective potentials of algal metabolites and highlight the recent progress in algae-based drug discovery. Finally, the rational strategy for algal compounds-based drug development has been discussed.

## 2. Pathophysiology of Brain Disorders

### 2.1. Neurodegenerative Disorders (AD and PD)

Neurodegenerative disorders, including AD and PD, are of major public health concern and contribute to the prime causes of dementia among elderly people. The pathological hallmarks of AD include extracellular deposition of amyloid plaque and intraneuronal aggregation of neurofibrillary tangles (NFT) [37]. On the other hand, PD is characterized by the degeneration of dopaminergic neurons in the substantia nigra [37] with the pathological hallmark of intraneuronal aggregation of *α*-synuclein [38]. Although the exact pathophysiology of these brain disorders remains elusive, it has been demonstrated that OS, neuroinflammation, mitochondrial dysfunction, and protein misfolding largely contribute to their development [37]. OS and neuroinflammation are two considerably diverse disease processes in many pathological events [39]. Conversely, they are interplayed with each other in the entire disease process. Thus, inhibition of neuroinflammation may reduce the OS and vice versa.

Oxidative stress (OS) is a pathological condition that develops when the production of reactive oxygen species (ROS) reaches an excessive level with lower efficiency of the cellular antioxidant defense system [40]. Factors contributing to OS in the brain include excitotoxicity, depletion of the cellular antioxidant system, high susceptibility to lipid peroxidation, and high oxygen demand [41]. OS may lead to mitochondrial dysfunction, which further results in the excessive ROS generation and establishes a vicious cycle of OS [42,43]. Moreover, the endoplasmic reticulum (ER), a site for protein folding, also takes part in ROS generation [44]. Protein misfolding in ER results in ER stress that is further responsible for ROS production [45]. ROS potentially contributes to the damage of cells through compromising the structure and function of biomolecules, including lipid peroxidation, protein oxidation, and deoxyribonucleic acid (DNA) damage, which eventually install neurodegeneration [38].

Neuroinflammation is another inevitable pathogenic factor of many neurodegenerative disorders [46]. Microglial activation is the major contributor to neuroinflammation [46]. A range of stimuli, including infection, trauma, toxic insults, and ischemia, may initiate microglial activation and disrupt the central nervous system (CNS) homeostasis [47,48]. Once activated, microglia released pro-inflammatory and neurotoxic elements, like chemokines, cytokines, proteases, eicosanoids, ROS, and excitatory amino acids [47]. All of these elements are documented as a key player in neuroinflammation-associated OS as well as chronic neurodegeneration [49]. The deposition of misfolded proteins, as evident in the major NDD, can also induce an inflammatory response, which further causes OS [50].

Dysregulation of cholesterol homeostasis is also a critical factor that could induce OS and inflammation, and thus may contribute to the pathogenesis of major brain disorders [51]. This disturbance in cholesterol metabolism in the brain is under the regulation of a cholesterol transport mechanism. Liver X receptor beta (LXR-β), once activated, promotes multiple genes that regulate reverse cholesterol transport and thus confers neuroprotection [52,53]. For instance, LXR-β agonist enhanced survival of dopaminergic neurons [54] and reduced the burden of mutant huntingtin [55] as well as promoted amyloid β (Aβ) clearance [56]. With the significant evidence of the implication of OS, neuroinflammation, and cholesterol dyshomeostasis in the pathobiology of neurodegenerative disorders, these pathological factors could be targeted for the development of potential therapeutics.

### 2.2. Ischemic Stroke

Ischemic stroke is responsible for the second-highest number of deaths and disability around the world [57]. It is a pathological condition resulting from sudden occlusion of blood supply to the brain. If the patient survives, the affected brain areas accompany the secondary damage due to the restoration of blood flow and reoxygenation. This ischemia/reperfusion (I/R) event initiates mitochondrial ROS generation [58] and subsequent inflammatory response [59].

Mitochondrial ROS is not only a crucial early driver of acute damage but is also considered an initiator of the consequence of a series of pathological features that develop over time following the reperfusion [60]. Initially, upon reperfusion, the burst of ROS production results in oxidative damage to mitochondria, and thereby disrupts ATP production [61], which ultimately initiates neuronal cell death cascades [62]. ROS-mediated mitochondrial damage further installs the inflammatory response via the activation of microglia and astrocytes as well as an influx of immune cells recruited by cytokines, adhesion molecules, and chemokines across the activated cerebral blood vessels [63]. This activation of the innate immunity triggers nuclear factor-kappa-B (NF-κB)-mediated production of numerous inflammatory cytokines that contribute to I / R injury [64]. Therefore, targeting OS and inflammatory response could be imperative to develop novel therapeutic strategies for the management of stroke.

### 2.3. Traumatic Brain Injury

Traumatic brain injury (TBI), an acquired brain injury caused by an external force or shock, is also considered to be a major cause of death globally, particularly in countries with a frequent incidence of traffic accidents [65]. Despite significant medical advances in recent times, the clinical outcomes of severely head-injured patients are not satisfactory.

As in ischemic stroke, mechanisms underlying the damages to the brain tissue with TBI are categorized into two classes: primary and secondary damages. Primary damage that irreversibly involves the mechanical damage of the skull and the brain has been complicated following the brain contusions, rupturing blood vessels, axonal injuries, and intracranial hemorrhages [66], whereas the secondary damage causes neuronal degeneration over time due to various biochemical changes such as OS, excitotoxicity, inflammation, and mitochondrial dysfunction [67]. Following TBI, various OS markers such as lipid peroxidation products, oxidized protein moieties, and DNA damage products accumulate in the brain while antioxidants and enzymes molecules such as glutathione (GSH), glutathione peroxidase (GPx), glutathione reductase (GR), glutathione S-transferases (GST), superoxide dismutase (SOD), and catalase (CAT) markedly decline [68]. It is suggested that treatment modalities associated with conferring neuroprotection on injured brain tissue and regeneration at the recovery stage of injured neurons have greater promise to restore at the site of brain injury following TBI.

## 3. Neuropharmacological Potentials of Marine Algae and Their Metabolites: Evidence from In Vitro Studies

**Figure 1:**
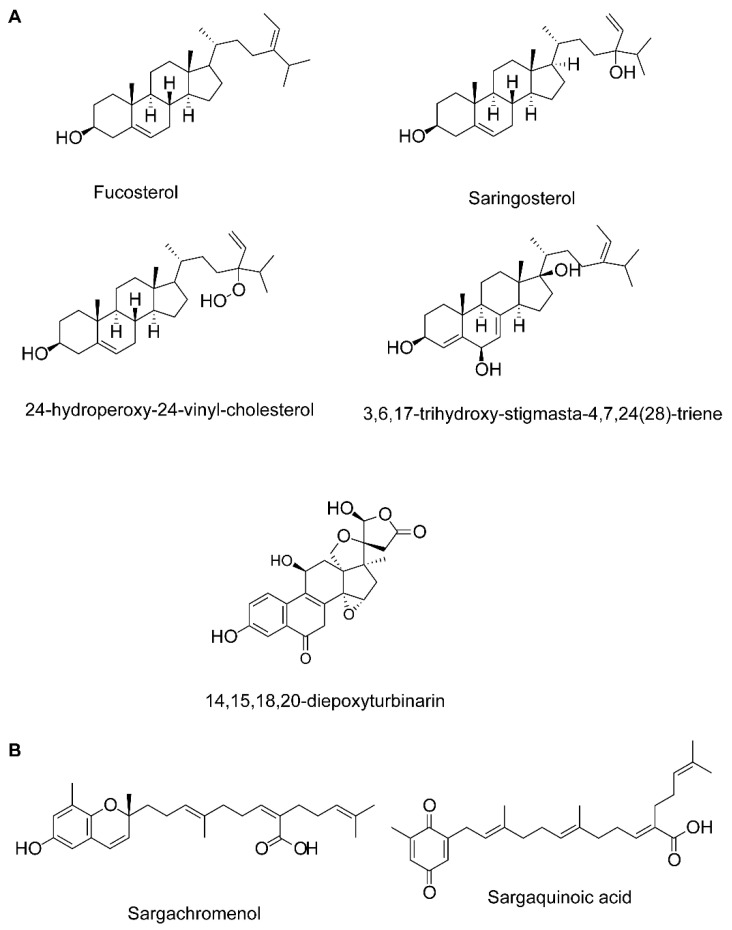
Chemical structure of sterols (**A**) and plastoquinones (**B**) of marine algae.

**Figure 2:**
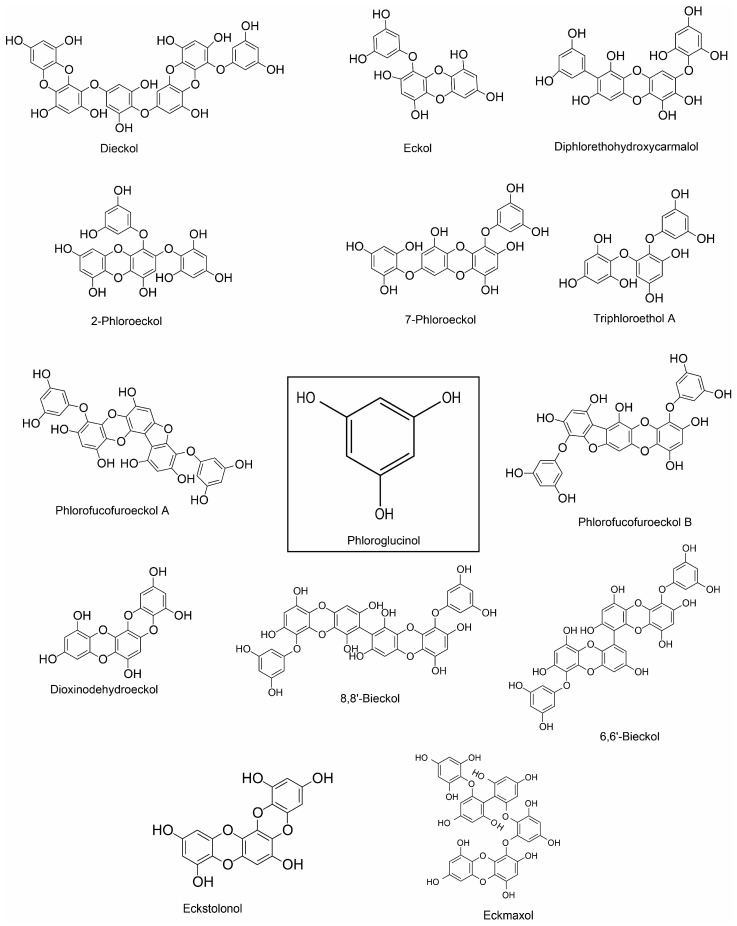
Chemical structure of phlorotannin of marine algae.

**Figure 3:**
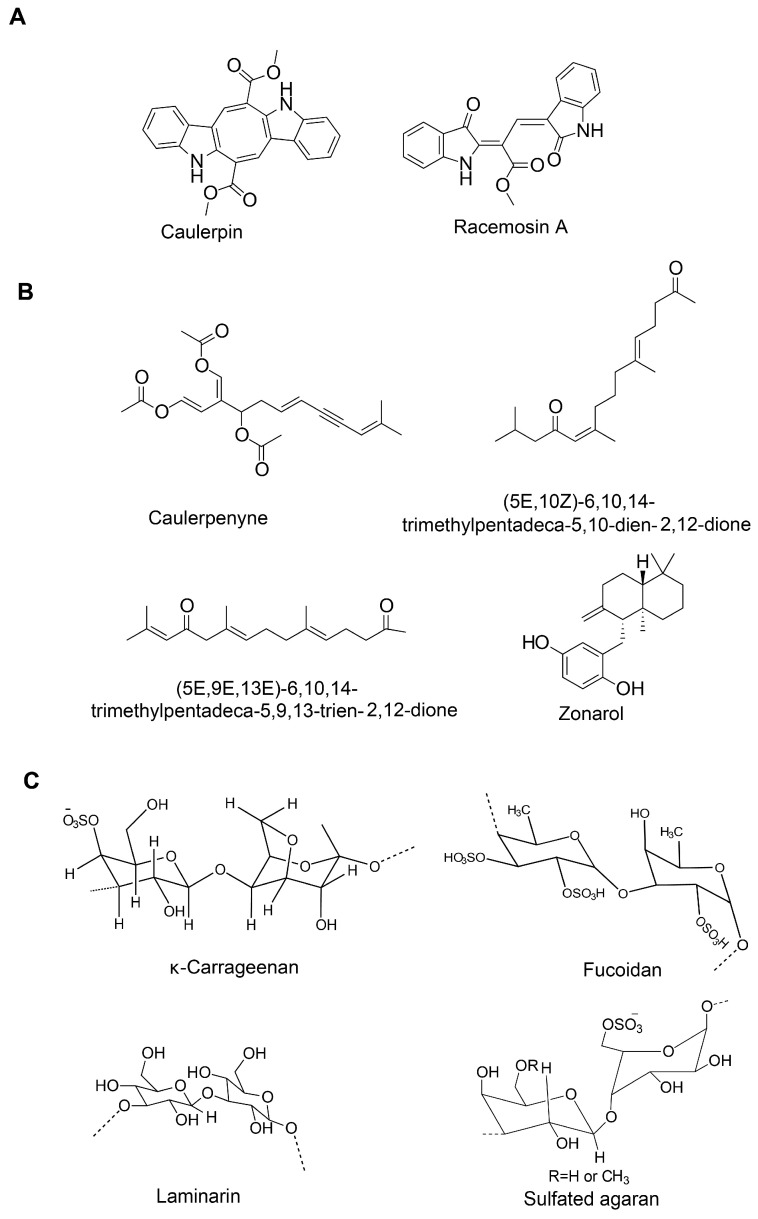
Chemical structure of alkaloids (**A**), sesquiterpenes (**B**) and polysaccharides (**C**) of marine algae.

**Figure 4:**
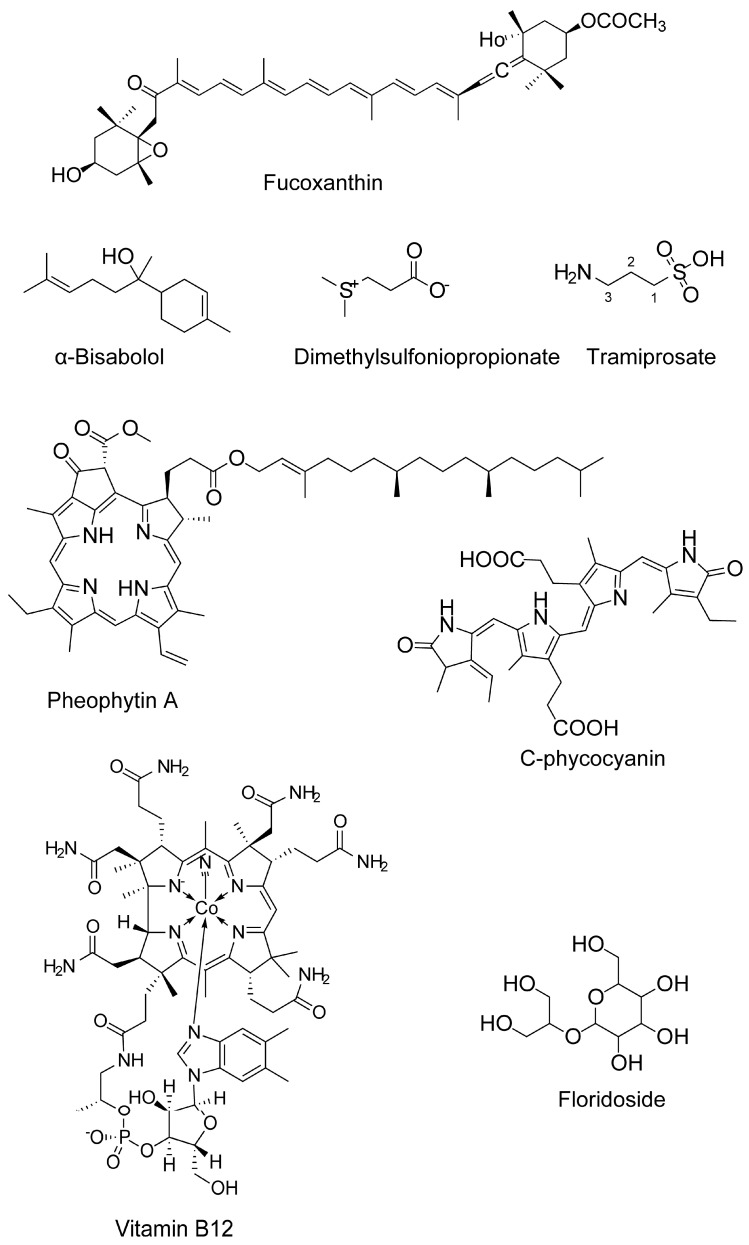
Chemical structure of miscellaneous compounds from marine algae.

Several compounds of diverse chemical classes have been reported from three major groups (brown, red, and green algae) of marine algae (Figure 1, Figure 2, Figure 3 and Figure 4). Neuropharmacological properties of these compounds reported in various in vitro models are compiled (Table 1) and discussed in the following subsections. Besides bioactive compounds, macroalgae that have shown promising neuroactive potentials, and thus demand further attention are also mentioned.

### 3.1. Antioxidant Activity

Marine algae-derived compounds have been reported to exhibit strong antioxidant property (Table 1), and thus may protect against oxidative damage. For example, fucoxanthin, a carotenoid from *Sargassum siliquastrum*, attenuated OS-induced DNA damage [69]. Fucoxanthin also prevented H_2_O_2_-induced DNA damage, which was associated with increased production of GSH, and expression of SOD [36]. Moreover, fucoxanthin promoted antioxidant defense in lipopolysaccharide (LPS)-activated BV-2 microglia by activating nuclear factor erythroid 2-related factor 2 (Nrf2)/heme oxygenase-1 (HO-1) pathway and cell survival through activating cAMP-dependent protein kinase (PKA)/cAMP response element-binding (CREB) pathway and increasing BDNF secretion [70]. Fucosterol raised cellular antioxidant enzymes, such as SOD, GPx, and CAT in experimental rats [71]. Jung and colleagues demonstrated that fucosterol prevented ROS production in tert-butyl hydroperoxide (t-BHP)-induced RAW264.7 macrophages [72]. In addition, fucosterol conferred protection from oxidative damage in HepG2 cells by raising the GSH level [73] and in lung epithelial cells by increasing the expression of SOD, CAT, and HO-1, and nuclear translocation of Nrf2 [74]. Glycoprotein of *U. pinnatifida* improved SOD activity (53.45%) and inhibited xanthine oxidase (Xox) activity (82.05%) [75]. Diphlorethohydroxycarmalol and 6,6′-bieckol from Ishige okamurae exhibited antioxidant activity and reduced intracellular ROS level in RAW264.7 cells [76]. Sulfated polysaccharide fractions from Porphyra haitanesis showed antioxidant activity and inhibited Lipid peroxidation in rat liver microsome [77]. Porphyran from Porphyra yezoensis showed superoxide anion and hydroxyl radical scavenging activity [78].

In addition, a great number of marine algae have shown antioxidant activity, including *Sargassum polycystum* and *Laurencia obtusa* [79], *Gelidium foliaceum*, and *Codium duthieae* [80], to mention a few.

### 3.2. Anti-Inflammatory Activity

An appreciable number of algal compounds have been reported for anti-inflammatory activity (Table 1). Fucoxanthin, a common carotenoid of brown algae, attenuated inflammation, and OS in glial cells [36,70]. In Aβ42-induced BV2 cells, fucoxanthin attenuated inflammatory response, which was manifested by decreased secretion of proinflammatory mediators, such as tumor necrosis factor-alpha (TNF-α), interleukin (IL)-6, IL-1β and prostaglandin E_2_ (PGE_2_) and reduced expression of inducible nitric oxide synthase (iNOS) and cyclooxygenase-2 (COX-2), and by lowering the phosphorylation of mitogen-activated protein kinase (MAPK) pathway [36]. In LPS-activated BV-2 microglia, fucoxanthin protected against neuroinflammation by lowering the expression of iNOS and COX-2 and reducing the secretion of inflammatory factors such as TNF-α, IL-6, PGE_2_, and nitric oxide (NO) that involved inhibition of protein kinase B (Akt)/NF-κB and MAPKs/ activating protein-1 (AP-1) pathways [70].

The anti-inflammatory activity of fucosterol has recently been reviewed [81]. In brief, fucosterol exhibited anti-inflammatory action [82] and attenuated LPS-induced inflammation in RAW 264.7 macrophage [72]; [83] and alveolar macrophage [84]. Fucosterol also protected against LPS- or Aβ-mediated neuroinflammation in activated microglial cells [85]. Several phlorotannins, such as dieckol [86], phlorofucofuroeckol A [87] and phlorofucofuroeckol B [88], 6,6’-bieckol [89], and 8,8’-bieckol [90] isolated from *Ecklonia* spp have been reported for their anti-inflammatory activities that involved suppression of NF-κB and MAPK pathways.

Algal polysaccharides are known to act as anti-inflammatory agents [91]. Fucoidan, a sulfated polysaccharide attenuated inflammatory response in LPS-stimulated BV2 microglia by suppressing NF-κB and extracellular signal-regulated kinases (ERK)/MAPK/Akt pathways [92]. In another study, fucoidan decreased the generation ROS and TNF-α in LPS-induced primary microglia [93]. κ-Carrageenan oligosaccharides and its desulfated derivatives from red algae attenuated TNF-α production and showed anti-inflammatory activity in LPS-activated microglia [94]. Porphyran from *Porphyra yezoensis* attenuated nitric oxide (NO) generation in LPS-stimulated RAW264.7 cells by suppressing iNOS expression [78,95]. Treatment with sulfated oligosaccharides of *Ulva lactuca* and *Enteromorpha prolifera* reduced inflammatory factors and downregulated the expression of p53 and fork-head box protein O1 (FOXO1) genes and upregulated the expression of Sirt1 gene in SAMP8 mice [96]. Alginate-derived oligosaccharide inhibited the expression of inflammatory enzymes and secretion of proinflammatory cytokines in LPS/Aβ-induced BV2 microglia. This oligosaccharide also reduced the expression of toll-like receptor 4 (TLR4) and NF-κB [97]. Priming of LPS-stimulated primary microglia and astrocytes with seleno-polymannuronate (Se-PM) reduced the expression of inflammatory enzymes and the production of inflammatory mediators by suppressing NF-κB and MAPK signaling [98]. Sargachromenol isolated from *Sargassum micracanthum* attenuated inflammatory response in LPS-induced RAW 264.7 macrophages [99]. Kang and colleagues reported that sargaquinoic acid of *Sargassum siliquastrum* suppressed inflammatory response in LPS-stimulated RAW 264.7 macrophages by downregulating NF-κB and c-JNK pathways [100]. Pretreatment of LPS-stimulated BV-2 microglial cells with floridoside inhibited inflammation by blocking p38/ERK phosphorylation [101]. Glycoprotein from *U. pinnatifida* (UPGP) reduced the expression of inflammatory enzymes and NO synthesis in LPS-stimulated RAW 264.7 macrophage [75]. Moreover, several algal alkaloids such as caulerpin, racemosin A-C, and caulersin were shown to have anti-inflammatory activity [102].

In addition, several marine algae have been reported to show anti-inflammatory properties in various experimental models, for instance, *Ecklonia cava* [103], *Myagropsis myagroides* [104,105], *Sargassum serratifolium* [106], and three Malaysian seaweeds (*Padina australis, Sargassum polycystum,* and *Caulerpa racemosa*) [107] in LPS-stimulated murine BV2 microglia; *Ulva conglobata* in interferon gamma-induced BV2 cells [108]; *Sargassum fulvellum* [109], *Sargassum horneri* [110], *Myagropsis myagroides* [111,112] in LPS-stimulated RAW 264.7 macrophage cells and *Sargassum serratifolium* in LPS-stimulated mouse peritoneal macrophages [113]. Owing to their capacity to modulate various inflammatory pathways, these algae and their respective compounds have shown encouraging effects in protecting various cell types from the inflammatory response.

### 3.3. Anticholinesterase Activity

Currently prescribed anti-AD drugs are mostly based on the inhibition of cholinesterase activity. Several algal metabolites have been reported to inhibit cholinesterase activity (Table 1). For example, fucosterol and 24-hydroperoxy 24-vinylcholesterol isolated from *E. stolonifera* showed inhibitory activity against butyrylcholinesterase (BChE) [114]. Another study also demonstrated anticholinesterase activity of fucosterol [85]. Enzyme kinetics and computational analysis indicated a non-competitive mode of acetylcholinesterase (AChE) inhibition of fucosterol [115].

Fucoxanthin exhibited anti-BChE activity which was of mixed inhibition type [116], whereas Lin and colleagues demonstrated that fucoxanthin showed non-competitive inhibition against AChE [117]. α-Bisabolol from *Padina gymnospora* showed inhibition against cholinesterase activity [118]. *U. pinnatifida*-derived glycoprotein showed AChE and BChE inhibitory activities [75].

The IC_50_ values for phloroglucinol, dibenzo [1,4] dioxine-2,4,7,9-tetraol and eckol from *Ecklonia maxima* range from 76.70 to 579.32 μM, with later two compounds possessing the highest AChE inhibitory activity [119]. Dieckol and phlorofucofuroeckol exhibited a similar anti-AChE activity [120]. Sargaquinoic acid and sargachromenol from *Sargassum sagamianum* have shown reasonable AChE inhibitory activity while the BChE inhibitory activity of sargaquinoic acid is 1000-fold higher than for AChE [121]. Tyrosol and its derivative, 4-(1,2-dihydroxyethyl) phenol from *Macrocystis angustifolia* showed anti-AChE activity [122]. Meroterpenoids, such as sargahydroquinoic acid, sargachromenol, and sargaquinoic acid of *S. serratifolium* exhibited potent anti-AChE activity [123]. Among the phlorotannins tested 8,8′-bieckol showed potent anti-AChE activity [124].

In addition, the extracts from some marine algae have shown anti-cholinesterase properties. These include *Halimeda cuneata* [80], *Padina australis* [125], *Botryococcus braunii* and *Nannochloropsis oculata* [126], *Cystoseira tamariscifolia* and *Cystoseira nodicaulis* [127], *Ishige foliacea* [128], and *Asparagopsis taxiformis* [129].

### 3.4. Anti-Amyloidogenic and Aggregation Inhibition Activity

As amyloid-β deposition is one of the hallmarks of AD, compounds that interfere with the generation of pathogenic Aβ and/or that inhibit its aggregation are of therapeutic importance. Several metabolites of marine algae have shown anti-amyloidogenic potentials (Table 1). For example, fucoxanthin at variant concentrations reduced the formation of Aβ_1–42_ fibril and Aβ1–42 oligomers, when co-incubated with Aβ_1–42_ monomers [135,136]. Both studies also demonstrated that fucoxanthin has been shown to inhibit Aβ aggregation [135,136]. Inhibition of β-site amyloid precursor protein cleaving enzyme 1 (BACE1) with fucoxanthin was of a mixed-type [134]. In addition, molecular docking analysis revealed a differential pattern of interaction [134]. Fucosterol showed a potential anti-BACE1 activity, which was a noncompetitive type [134]. Supporting these findings, a recent in silico study also explained the binding and interaction pattern of fucosterol with BACE1 [142]. α-Bisabolol from *Padina gymnospora* prevented oligomers formation as well as disaggregated the matured fibrils [118]. Glycoprotein from *U. pinnatifida* exhibited anti-BACE1 activities with IC_50_ values of 73.35 ± 2.54 μg/mL [75]. Meroterpenoids, such as sargahydroquinoic acid, sargachromenol, and sargaquinoic acid of *S. serratifolium*, exhibited potent anti-BACE1 activity [123]. Phlorotannins, such as eckol, dieckol, and 8,8′-bieckol from *Ecklonia cava* showed anti-BACE1 activity [124]. Olasehinde et al. reported that four South African macroalgae such as *Gracilaria gracilis, Ulva lactuca, Ecklonia maxima*, and *Gelidium pristoides* exhibited anti-cholinesterase, anti-BACE1, and Aβ aggregation inhibitory activities, indicating that these types of seaweed could be potential sources of anti-AD agents [35]. *Ishige foliacea* extract showed β-secretase inhibition property [128].

### 3.5. Cholesterol Homeostasis and Aβ Clearance Activity

Some algal metabolites are known to activate LXR-β (Table 1), and thus help regulate cholesterol homeostasis and enhance Aβ clearance [56]. Fucosterol is a selective LXR-β agonist that upregulated several LXR target genes, such as *ATP-binding cassette transporter A1* (*ABCA1*), *ABCG1*, and *apolipoprotein E* (*ApoE*) [138,139], suggesting that fucosterol could play a significant role in brain cholesterol homeostasis. Saringasterol, another selective LXR-β agonist isolated from *S. fusiforme*, activated the expression of similar LXR target genes in multiple cell lines [139]. Alginate-derived oligosaccharide isolated from marine brown algae promoted the microglial phagocytosis of Aβ, which is connected to the activation of toll-like receptor signaling [97]. As cholesterol imbalance and impaired protein clearance system significantly contribute to the pathogenesis of major neurological disorders, more efforts should, therefore, be paid to explore similar compounds that may help regulate cholesterol homeostasis and proteostasis.

### 3.6. Monoamine Oxidase Inhibition and Affinity to Dopaminergic Receptors

Inhibition of MAO-A (monoamine oxidase-A), an enzyme that catalyzes oxidative deamination of neuroamines, such as dopamine, norepinephrine, and serotonin (5-HT), is a putative approach to raise the brain 5-HT level, thus alleviating the symptoms of Parkinsonism [143]. Seong and team screened the multi-target effects of three phlorotannins, i.e., phloroglucinol, phlorofucofuroeckol-A (PFF-A), and dieckol against human MAO-A and -B and various neuronal G-protein-coupled receptors (GPCRs). Of these, PFF-A exhibited a relatively higher inhibition against both *h*MAO isoforms, with greater selectivity toward *h*MAO-B (Table 1). Enzyme kinetics and computational findings indicated that PFF-A noncompetitively interacted with *h*MAOs and acted allosterically. In a functional assay for GPCR screening, dieckol and PFF-A showed a multi-target combination of D_3_R/D_4_R agonism and D_1_/5HT_1A_/NK_1_ antagonism [140].

### 3.7. Anti-Aging

Algal compounds that exhibited anti-aging effects (Table 1) could have therapeutic value for physiological as well as pathological brain aging. Sulfated oligosaccharides of *Ulva lactuca* and *Enteromorpha prolifera*, when treated in SAMP8 mice, increased the serum level of antioxidant molecules and total antioxidant capacity, and decreased the levels of malondialdehyde (MDA) and advanced glycation end products in the serum of experimental mice [96]. It has also been observed that these oligosaccharides decreased inflammatory factors, increased BDNF and choline acetyltransferase (ChAT) levels, and promoted the survival of hippocampal neurons. The underlying mechanisms involved the downregulation of *p53* and *FOXO1* genes and the upregulation of *Sirt1* gene [96]. *Caenorhabditis elegans*, when treated with fucosterol (at 50 µg/mL), survived longer compared to control, indicating that this algal compound might help extend life-span and thus might protect against premature aging [141]. Antioxidant, anti-inflammatory, and immunostimulatory properties of fucosterol were supposed to be involved in its pro-survival effect [144].

### 3.8. Neurotrophic Activity

Compounds with neuritogenic potentials are promising to reconstruct a damaged neuronal network, which is a characteristic feature of neurodegeneration. Several algal metabolites have shown a promising neurite outgrowth promoting potentials in cell culture conditions (Table 2). Sargachromenol from *Sargassum macrocarpum* promoted nerve growth factor (NGF)-dependent neuronal differentiation of PC12D cells by activating cyclic AMP-mediated protein kinase and MAPK1/2 and supported their survival by activating phosphatidylinositol-3 kinase (PI3K) [145]. Sargaquinoic acid, another metabolite from *S. macrocarpum*, promoted neuritogenesis in PC12D cells, which involved cooperation between two independent pathways, i.e., the TrkA-MAPK pathway and adenylate cyclase-PKA pathway [146]. Ina and colleagues demonstrated that the neurodifferentiation of PC12 cells by pheophytin a of *Sargassum fulvellum* required the presence of NGF and involved the activation of an MAPK signaling pathway [147]. Vitamin B_12_, a chlorophyll-related analog to pheophytin a, also stimulated NGF-dependent PC12 cell differentiation by an MAPK signaling pathway [148].

Dimethylsulfoniopropionate (DMSP) promoted neurite outgrowth and protected against TDA-induced cytotoxicity, involving the upregulation of Hsp32 and activation of the extracellular signal-regulated kinases 1/2 (ERK1/2) [149]. Fucoxanthin has shown to exhibit neurite outgrowth activity (15.7–31% of cells to develop neurite outgrowth) at much lower concentrations (0.1–2 μM), in the absence of NGF support, indicating that this marine carotenoid could a potential neurotrophic molecule [136]. *Gracilariopsis chorda* and its active compound arachidonic acid modulated spine dynamics, and potentiated functional synaptic plasticity of hippocampal neurons [150].

In addition, several marine algae have shown to promote neurite outgrowth. For example, *Sargassum macrocarpum* and *Jania adharens* showed neuritogenic potentials and promoted neuron-specific dendrites and axons from PC12D cells [151]. Two compounds, namely sargachromenol [145] and sargaquinoic acid [148], having neurite outgrowth potential were already isolated. *Porphyra yezoensis* and its compound taurine facilitated neuronal development and maturation of primary hippocampal neurons [152]. *Gelidium amansii* [153,154,155,156], *Sargassum fulvellum* [157], *Undaria pinnatifida* and *Saccharina japonica* [158], *Gracilariopsis chorda* [150,159], and carrageenophyte *Kappaphycus alvarezii* [160,161,162,163] promoted neuronal morphology and functions. Of these, *G. amansii* that exhibited neuromodulatory potentials in several studies [153,154,155,156] could be the most promising candidate for further isolation of neurotrophic agents and thus expects special attention of natural product chemists.

### 3.9. Neuroprotective Activity

Compounds that possess antioxidant, anti-inflammatory, anti-amyloidogenic, and anti-aggregation, cholesterol homeostasis, and protein clearance activities are expected to show potential neuroprotective effects. Congruently, the following metabolites isolated from marine algae have been reported to confer neuroprotection against a range of toxic stimuli (Table 3).

Several studies reported the neuroprotective activity of fucoxanthin. For example, fucoxanthin attenuated β-amyloid oligomer-induced [164] and H_2_O_2_-induced [165] apoptosis and OS in SH-SY5Y cells through activating a pro-survival PI3K/Akt pathway and suppressing the proapoptotic ERK pathway. Fucoxanthin-mediated protection against H_2_O_2_-induced apoptosis in primary cerebellar granule neurons also involved a similar neuroprotective mechanism [165]. Co-incubation of fucoxanthin with Aβ1–42 oligomers formed modified Aβ1–42 oligomers, which were relatively less toxic to SH-SY5Y cells compared to Aβ1–42 oligomers, indicating that fucoxanthin-triggered structural modification of Aβ1–42 oligomers reduced their neurotoxicity [135]. Fucoxanthin, isolated from *Undaria pinnatifida,* also attenuated hypoxia/reoxygenation (H/R)-induced cellular injury in primary cortical [166] and hippocampal neurons [167]. Likewise, fucoxanthin suppressed oxygen-glucose deprivation/ reperfusion (OGD/R)-induced neuronal apoptosis, via activating the Nrf2/HO-1 signaling [168]. In the TBI model of mouse primary cortical neurons, fucoxanthin promoted neuronal survival against secondary injury and enhanced antioxidant enzymes such as HO-1 and NAD(P)H dehydrogenase [quinone] 1 (NQO-1) via activating Nrf2-ARE and Nrf2-autophagy pathways [169]. Fucoxanthin also attenuated both Aβ1-42- and H_2_O_2_-induced toxicity in PC12 cells [136].

Zonarol (ZO), a *para*-hydroquinone-type molecule from *Dictyopteris undulata* protected against OS in HT22 hippocampal and cerebrocortical neurons by activating the Nrf2/ARE pathway [170]. It induced the expression of NQO-1, HO-1, and peroxiredoxin 4 (PRDX4) and thus helps regulate intracellular redox state [170]. α-Bisabolol, an active compound of *Padina gymnospora*, protected against Aβ25-35-induced neurotoxicity in PC12 cells [137] and also in Neuro2a cells and transgenic *C. elegans* [171]. In PC12 cells, the rescuing effects of α-bisabolol against Aβ induced neurotoxicity were similar to donepezil, which is a currently prescribed anti-AD drug [137]. In Neuro2a cells, α-bisabolol exhibited inhibition against cholinesterase and β-secretase activity. In addition, α-bisabolol prevented apoptosis in Neuro2a cells by inhibiting the production of ROS and reactive nitrogen species (RNS) and reducing the expression of bcl-2-like protein (Bax) and caspase-3 [171]. In a transgenic *C. elegans* Alzheimer’s model, α-bisabolol attenuated Aβ-induced proteotoxicity by decreasing the expression of angiotensin-converting enzyme 1 (ace-1), hsp-4, and Aβ [171]. The neuroprotective roles of fucosterol have been reviewed in our recent article [81]. In brief, fucosterol attenuated Aβ-induced neurotoxicity in hippocampal neurons [172] and SH-SY5Y cells [173]. In addition, three isolated compounds including α-tocospirone, (23E)-3β-hydroxy-stigmasta-5,23-dien-28-one and (22E)-3β-hydroxy-cholesta-5,22-dien-24-one from Caulerpa racemose attenuated Aβ25-35-induced toxicity in SHSY5Y cells [174].

Phlorotannins, a specialized group of tannins, particularly rich in brown algae, have shown significant neuroprotective effects in several neurotoxicity models. Liu and colleagues evaluated three phlorotannins, including 8,8’-bieckol, dieckol, and eckol for their neuroprotection against Aβ25-35-mediated cytotoxicity in PC12 cells [96]. Of these, dieckol showed maximum protection, although all were shown to suppress inflammatory response by inactivating the NF-κB pathway [96]. A similar study by Ahn and teams demonstrated that six phlorotannins, such as phloroglucinol, dioxinodehydroeckol, eckol, dieckol, phlorofucofuroeckol A, and 7-phloroeckol from *Eisenia bicyclis* protected against Aβ-induced cytotoxicity by inhibiting ROS generation and Ca^2+^ release [175]. Dieckol attenuated glutamate-induced excitotoxicity in primary cortical neurons and HT22 neurons by scavenging ROS and nuclear factor-like 2/heme oxygenase-1 pathway [176]. In addition, in another study, phloroglucinol from *E. cava* suppressed Aβ1-42 -provoked ROS accumulation in an HT-22 hippocampal cell line [177].

Phloroglucinol also rescued the Aβ1-42-induced reduction of dendritic spine density and synaptic protein (synaptophysin and postsynaptic density) levels in primary cultures of rat hippocampal neuronal [177]. Kang and co-investigators isolated five phlorotannins, such as phloroglucinol, eckol, triphloroethol A, eckstolonol, and dieckol from *E. cava* that attenuated H_2_O_2_-induced oxidative damage in HT22 hippocampus neurons by lowering ROS production, lipid peroxidation and Ca^2+^ release [178]. Phlorofucofuroeckol attenuated glutamate-induced cytotoxicity and improved mitochondrial dysfunction in PC12 cells [180]. Preconditioned HT22 hippocampal neurons with diphlorethohydroxycarmalol (DPHC), a phlorotannin of *Ishige okamurae*, was able to escape H_2_O_2_-induced oxidative damage due to antiapoptotic, pro-survival, and antioxidant potentials of DPHC [179]. Eckmaxol, a phlorotannin of *Ecklonia maxima*, reduced Aβ-oligomer-induced neuronal apoptosis in SH-SY5Y cells by inhibiting GSK-3β and ERK pathways [181,203].

Several studies have confirmed the neuroprotective capacity of algal polysaccharides, including fucoidan [204] and carrageenan. Fucoidan, a sulfated polysaccharide, attenuated Aβ_1−42_-induced neurotoxicity in rat cholinergic basal forebrain neurons [182]. It restored Aβ-induced decline in whole-cell currents, increased phosphorylation of protein kinase C (PKC), and showed antioxidant and anti-apoptotic effects [182]. Fucoidan protected H_2_O_2_-induced cell death in PC-12 cells by activating the PI3K/Akt signaling pathway. The antioxidant, antiapoptotic, and prosurvival effects of fucoidan could explain its neuroprotection capacity [184]. Fucoidan protected against Aβ25-35 and d-Gal-induced neurotoxicity in PC12 cells by reducing OS, suppressing apoptosis pathway, and promoting antioxidant defense [185]. Wu and colleagues reported that fucoidan suppressed intracellular Ca^2+^ responses by selective inhibition of N-methyl-D-aspartate (NMDA) receptors in cortical neurons and L-type Ca^2+^ channels in hippocampal neurons [187]. Three fucoidan extracts from *Sargassum crassifolium* attenuated H_2_O_2_-induced cytotoxicity in rat pheochromocytoma PC-12 cells [205]. In the MPP^+^ PD model, fucoidan attenuated cytotoxicity in a dopaminergic neuronal precursor cell line (MN9D) [183,186] by protecting lysosomes, reducing the expression of light chain 3-II (LC3-II), inhibiting the expression of cathepsin D (CatD)-Bax and the OS response [186]. Fucoidan of *Sargassum hemiphyllum* attenuated 6-hydroxydopamine-induced apoptosis in SH-SY5Y cells [206]. The acidic oligosaccharide sugar chain attenuated Aβ-stimulated astrocytes conditioned medium-induced cytotoxicity in SH-SY5Y cells by mitigating oxidative damage, reducing inflammatory response, and preventing Ca^2+^ influx [189]. In addition, κ-carrageenan-derived pentasaccharide (KCP) protected against Aβ25-35-induced neurotoxicity in SH-SY5Y cells by regulating the c-Jun N-terminal kinase (JNK) signaling pathway [192]. Moreover, κ-carrageenan from *Hypnea musciformis* attenuated 6-hydroxydopamine-induced neurotoxicity on SH-SY5Y cells by modulation of the mitochondria transmembrane potential and reducing caspase 3 activity [207]. Oligo-porphyran (OP), an acid hydrolytic product of porphyran (a polysaccharide of *Pyropia yezoensis*) attenuated 6-OHDA-induced cytotoxicity in PC12 cells by activating PI3K/ Akt/PKC pathway that involved anti-apoptotic, antioxidant and anti-inflammatory signals [188].

Sargaquinoic acid identified from *Sargassum macrocarpum* promoted cell survival and neurite regeneration and attenuated H_2_O_2_-induced OS in PC12D cells [208]. Racemosin A, a bisindole alkaloid from *Caulerpa racemose,* attenuated Aβ25-35-induced damage in SH-SY5Y cells [190]. Tramiprosate, a small aminosulphonate compound of red marine algae, attenuated OGD- or NMDA-induced injury in PC12 cells and primary cortical neurons [191] by disrupting the interaction between PSD95 and nNOS and inhibition of nNOS translocation [191]. Potentials of tramiprosate against AD and PD have also been reviewed elsewhere [28,209,210]. Dimethylsulfoniopropionate protected against tropodithietic acid-induced cytotoxicity in OLN-93 and N2a cells by lowering the activation of ERK1/2 and induction of HSP32 [149]. Phycoerythrin-derived peptide isolated from *Pyropia yezoensis* promoted survivability of frontal cortical neuron by activating TrkB receptor-ERK1/2 signaling and attenuating ER stress in rat prefrontal cortex [211] and attenuated glutamate-induced ER stress and senescence of rat primary hippocampal neurons [212]. Stearic acid from *Caulerpa racemosa* protected against OGD-induced SH-SY5Y cell damage while (8E)-heptadec-8-en-7-one showed moderate neuroprotective activity against Aβ25-35-induced SHSY5Y cell damage [213].

In addition, extracts from several marine algae have shown neuroprotective activity in various in vitro models. The neuroprotective algae include *Ulva conglobata* that protected against glutamate-induced neurotoxicity in murine hippocampal HT22 cell line [108], *Botryococcus braunii*, and *Nannochloropsis oculata* against H_2_O_2_-induced cytotoxicity in dopaminergic SH-SY5Y cells [126], *Padina pavonica, Sargassum muticum, Saccorhiza polyschides,* *Codium tomentosum*, and *Ulva compressa* [214], and Bifurcaria bifurcata [215] against 6-hydroxidopamine-induced cytotoxicity in neuroblastoma cells, *Cystoseira tamariscifolia* and *Cystoseira nodicaulis* against H_2_O_2_-induced cytotoxicity in SH-SY5Y cells [127], *Gracilaria corticata* against aluminium-induced neurotoxicity in the hippocampus, and cerebral cortexes of rat brains [216], Australian macroalgae against Aβ 1-42-induced neurotoxicity in PC-12 cells [217], *Ishige foliacea* against H_2_O_2_- or Aβ-induced cell death in human neuroblastoma SH-SY5Y cells [128], *Undaria pinnatifida* against endoplasmic reticulum stress in hypothalamic neurons [218] and *Gracilariopsis corda* [219] and *Gelidium amansii* [153] against H/R-induced oxidative damage in primary hippocampal neurons, indicating that these algae could offer some potential compounds with encouraging neuroprotective activity, and, therefore, demand further investigation.

## 4. Neuropharmacological Potentials of Marine Algae and Their Metabolites: Evidence from In Vivo Studies

The neuroprotective effects of some potential algal compounds that were reported in the in vitro conditions have successfully been translated into animal models (Table 3), suggesting that these compounds could be potential candidates for further evaluation in the clinical trials.

Fucoidan is one of the algal compounds that has shown strong neuroprotection in several animal models. In the PD model of C57 / BL mice, fucoidan ameliorated MPTP-induced behavioral deficits, probably by elevating dopamine and its metabolite levels and increasing tyrosine hydroxylase expression [183]. In addition, fucoidan inhibited MPTP-induced lipid peroxidation and restored antioxidant capacity [183]. Similarly, fucoidan also improved behavioral capacity, by attenuating the loss of dopaminergic neurons and inhibited the deleterious activation of microglia in the substantia nigra pars compacta in LPS-induced neurotoxicity [93]. In an Aβ-induced rodent AD model, fucoidan ameliorated impaired memory, by reversing the decreased activity of ChAT, SOD, and GPx, increased activity of AChE, and rectifying the imbalance between apoptosis and pro-survival signals [193]. Fucoidan improved d-Gal-induced cognitive impairment in mice by mitigating OS and attenuating the caspase-dependent apoptosis pathway [185]. Wang and colleagues demonstrated that the supplementation of fucoidan alleviated Aβ-induced paralyzed phenotype in a transgenic *C. elegans* AD model [194]. Fucoidan reduced Aβ accumulation, probably by promoting proteasomal activity [194]. In another study, fucoidan-rich substances from *Ecklonia cava* improved trimethyltin-induced cognitive dysfunction by inhibiting Aβ production and Tau hyperphosphorylation [195]. Fucoidan also attenuated transient global cerebral ischemic injury in the gerbil hippocampal CA1 area through mitigating glial activation and oxidative stress [196].

Laminarin, another polysaccharide of brown algae, has shown to protect I/R injury in gerbil models. Intraperitoneal injection of laminarin (50 mg/kg) following 5 min I/R attenuated reactive gliosis (anti-inflammatory) in the hippocampal CA1 of young gerbils [197]. A similar study following the same experimental protocol, but with aged gerbils, showed that laminarin (50 mg/kg) attenuated ischemia-induced death of pyramidal neurons in the hippocampal CA1 of aged gerbils [198]. This neuroprotective effect of laminarin is attributed to its antioxidant and anti-inflammatory properties [198]. Oligo-porphyran, a synthetic product of porphyran (*Pyropia yezoensis*) ameliorated behavioral deficits in 6-OHDA-induced Parkinsonian mice model by protecting dopaminergic loss and activating the PI3K/Akt/Bcl-2 pathway that involved cellular signaling of anti-apoptosis and antioxidation [199]. Zhang and colleagues demonstrated that porphyran from *Pyropia haitanensis* improved the Aβ1-40-induced learning and memory deficits probably by elevating cerebral acetylcholine level [200].

Fucoxanthin is another significant algal metabolite that was found to be effective in a wide range of brain dysfunction (such as AD, ischemic stroke, and traumatic brain injury). Fucoxanthin ameliorated scopolamine-induced [135] and Aβ oligomer-induced [117] cognitive impairments in mice, possibly by inhibiting AChE activity and OS, modulating ChAT activity, and increasing BDNF expression. Fucoxanthin alleviated cerebral ischemic/reperfusion (I/R) injury, improved the neurologic deficit score, and downregulated the expression of apoptosis-linked proteins in brain samples [168]. Fucoxanthin also attenuated traumatic brain injury that involved the Nrf2-ARE and Nrf2-autophagy pathways-dependent neuroprotective mechanism [169].

Fucosterol co-infusion ameliorated sAβ1-42-induced cognitive deficits in aging rats by modulating BDNF signaling [172]. Dieckol and phlorofucofuroeckol raised the brain level of acetylcholine by inhibiting AChE and reduced the inhibition of latency in ethanol-intoxicated memory-impaired mice [120]. Yang and co-investigators demonstrated that stereotaxic injection of phloroglucinol promoted synaptic plasticity and improved memory impairment in 5XFAD (Tg6799) mice [177]. In a later study, the same group reported phloroglucinol (orally administered)-mediated amelioration of cognitive dysfunction that involved a reduction in the amyloid β peptide burden and pro-inflammatory mediators and restoration of reduction in the dendritic spine density in the hippocampus of 5XFAD mice [220]. Phlorofucofuroeckol improved ischemic brain damage in the rat MCAO model [180]. C-Phycocyanin improved the functional outcome and survival of gerbils on global cerebral I/R injury [201]. The in vitro neuroprotective effect of tramiprosate has been translated into in MCAO rat model in which it improved functional recovery following ischemic stroke [191]. Sulfated agaran, a sulfated polysaccharide from *Gracilaria cornea,* attenuated oxidative/nitrosative stress and ameliorates behavioral deficits in rat 6-hydroxydopamine Parkinson’s disease model [202]. It raised levels of dopamine, 3,4-Dihydroxyphenylacetic acid (DOPAC), GSH, and BDNF, decreased serotonin (5-HT) and thiobarbituric acid reactive substances (TBARS) levels, and decreased the expression of p65, IL-1β, and iNOS [202]. Glycoproteins isolated from *Capsosiphon fulvescens* ameliorated aging-induced spatial memory deficits by attenuating GSK-3β-mediated ER stress in rat dorsal hippocampus [221] and promoted probiotics-induced cognitive improvement in aged rat model [222]. *Gracilariopsis chorda* and its active compound arachidonic acid, given independently through oral route for 10 days, improved scopolamine-induced memory impairment in mice [150].

In addition, extracts from several marine algae have shown to either ameliorate memory impairment or enhance cognition in various in vivo models. For instance, *Gelidiella acerosa* attenuated Aβ25-35-induced cytotoxicity and memory deficits in mice [223], *Sargassum swartzii* improved memory functions in rats [224], *Ishige foliacea* [128], *Undaria pinnatifida* [225] ameliorated scopolamine-induced memory deficits in mice, *Haematococcus pluvialis* recovered Alzheimer’s disease in rats [226], and fermented *Spirulina maxima* prevented memory impairment in mice [227]. In addition, some marine algae have shown to attenuate ischemic injury in stroke models. For example, *Ecklonia cava* ameliorated transient focal ischemia in the rat MCAO model [228].

## 5. Recent Progress on the Development of Marine Algae-Based Neurotherapeutics

An algal oligosaccharide, sodium oligomannate, recently received conditional approval in China for improving cognitive function in patients with mild to moderate AD [32]. In preclinical studies, sodium oligomannate conferred neuroprotection against Aβ-induced neurotoxicity in human neuroblastoma cells [229] and ameliorated memory dysfunction in the 5XFAD transgenic mouse model [230]. Sodium oligomannate can cross the blood–brain barrier through glucose transporter (GLUT1) and inhibits Aβ fibril formation and destabilizes the preformed fibrils into nontoxic monomers [230]. Although the complete mechanism of pharmacological actions remains unclear, sodium oligomannate harnessed neuroinflammation and thus ameliorated memory impairment by suppressing gut dysbiosis and the associated phenylalanine/isoleucine accumulation [230]. In a phase IIa pilot study in patients with AD, there was an elevation of Aβ1–42 levels in the cerebrospinal fluid (CSF) following sodium oligomannate treatment, suggesting a significant role in Aβ clearance into CSF [231]. There was a differential reduction in the cerebral glucose metabolic rate (CMRglu) in various brain regions following sodium oligomannate in clinical trials [231]. While in a phase IIa trial, the CMRglu in left orbitofrontal gyrus, left precuneus, right posterior cingulate gyrus, and right hippocampus were found to be low, in a phase III trial, the lower rate was reported in superior parietal gyrus, inferior parietal gyrus, angular gyrus, and anterior wedge [232]. However, this newly approved drug lacks some advanced information like global data of effectivity and thus requires a large-scale global trial before it receives approval from the Food and Drug Administration (FDA).

## 6. Algal Metabolites-Based Drug Discovery and Design

While a significant quantity of active compounds has been isolated from marine algae and added to the compound databases [233,234,235,236,237,238] every year, it is disappointing that very few of them have access to clinical trial and the success rate is also not very satisfactory. In this context, the current strategy of drug development requires a reformation with the inclusion of some modern approaches, such as virtual screening and network pharmacology. The system biology approach along with an in silico study constitutes a potential computation tool that can better explain how a biologically effective compound interacts with the signal molecules of various cellular pathways.

Recent multitarget drugs have been designed by analyzing the 3D structure of already characterized compounds and crystal structure of target protein molecules. This information is focused on the virtual design of new chemical entities that include more than one biological function in a single molecule [239]. This approach is also known as target fishing, which identifies not only interacting proteins but also potential off-targets, and thus helps to understand polypharmacology, pharmacokinetics, and toxicity in the early stages of drug discovery [240]. For example, using in silico target fishing approach, Hannan and colleagues elucidated pharmacological mechanism of fucosterol-mediated neuroprotection and demonstrated that fucosterol showed interaction with potential targets, including LXR, TrkB, GR, Toll-like receptor (TLR) 2/4, and BACE1 [142]. Computational methods involving target screening are classified based on their principle including pharmacophore screening, shape screening, and reverse docking. When the target is available in the crystal structure, target fishing can be accomplished by a reverse docking approach, while, in the target’s absence, pharmacophore or shape screening can be used to find the relevant target by comparing pharmacophoric feature or shape of the compound, taking information from protein–ligand binding databases [241]. In this effort, several natural product databases containing compound target interactomes are available nowadays including, SuperNatural [242], TCMID [243], TCMSP [244], and many others [245,246]; however, not many are dedicated to marine algae [233,234,235]. Although algal metabolites show structural diversity and redundancy, the mentioned databases could still be available for network pharmacology to get insight into the disease-modifying mechanisms. Following this in silico approach, Vitale et al. identified caulerpin as a PPAR agonist which was confirmed by both in vitro and in vivo assays [247]. In a reverse way, virtual screening through molecular docking analysis could be an alternative to find out potent hits from a large chemical library for a single target.

Compared to experimental high throughput screening, virtual screening, either by ligand or structure-based approach, can deliver the shorten cycle of hit discovery, with higher success hit rates. Furthermore, a structure-based approach consisting of molecular docking, receptor-based pharmacophore modeling together with molecular dynamics simulations and MM/PB(GB)SA approaches not only predict protein–ligand interaction but also provide a detailed binding mechanism, protein dynamics, and also highlight structure–activity relationship (SAR) for future drug design [248]. Several recent studies have been adopting molecular docking techniques to analyze detailed protein–ligand interaction for marine bioactive compounds. For example, Jung et al. employed molecular docking studies to predict comparative binding interaction of monoamine oxidase (MAO) with fucoxanthin, a carotenoid from *Eisenia bicyclis*, where they revealed fucoxanthin as a reversible competitive hMAO inhibitor, binding strongly to the enzyme, following hydrogen bonding and hydrophobic interactions [249]. A similar approach has been applied to elucidate the interaction of fucosterol and fucoxanthin with BACE1 while analyzing BACE1 enzyme inhibition by fucosterol and fucoxanthin. Here, binding interaction analysis by molecular docking identified that the presence of hydroxyl group in fucosterol and fucoxanthin is important for BACE1 inhibition, by which both compounds interacted with Lys224 residue, Gly11, and Ala127 of the active site, respectively [134]. Interestingly, fucoxanthin was also identified as a dopamine agonist, where a molecular docking study suggested that it formed H-bonding with Ser196 and Asp115 of the D4 receptor, and Ser196 and Thr115 residues of D3 receptors [250]. The same group also identified some bromophenols derivatives as D3R and hD4R antagonists and studied the interaction and binding pattern by molecular docking [251].

In addition, several studies employed virtual screening to identify potent lead molecules from the database of seaweed metabolites. For instance, Florest et al. identified sigma-2 (σ_2_) receptor binding ligand by using both structure and ligand-based screening [252]. However, less effort has been deployed to develop marine natural product libraries, although significant studies so far have reported many compounds isolated from marine sources by large populations in the world. In this exertion, Davis et al. developed a chemical library of the natural compounds from marine algae, SWMD, comprised of 1110 metabolites, isolated from brown algae (266), green algae (33), and red algae (811) along with their physical and chemical properties [233]. Nevertheless, the information including experimentally-determined quantitative activity data and source information for more marine algal metabolites is still needed to facilitate computational based approaches in the exploration of marine compounds for future drug discovery.

## 7. Safety Issues on Marine Algae-Derived Compounds

As a popular food material in East Asian countries, including Japan, Korea and China, seaweed is consumed without reported toxicity. However, the concern is that seaweed may sometimes accumulate a considerable amount of heavy metals, such as cadmium, arsenic, mercury, and lead, and even some essential microelements such as iodine and iron [253]. It is, therefore, essential to conduct appropriate safety evaluations for seaweed. More importantly, while there are safety concerns during therapeutic development, the toxicity profile of seaweed-derived compounds needs to be thoroughly investigated. Safety information on algal metabolites is limited. However, toxicity profiles of algal polysaccharides have been reported by several studies. Observations from both in vitro and in vivo studies satisfied the non-toxic behavior of fucoidan irrespective of algal sources [254]. Fucoidan isolated from *Undaria pinnatifida* and *Laminaria japonica* was found to be safe in animal models given at very high oral doses [255,256,257,258]. Clinical studies also demonstrated the non-toxic health benefits of fucoidan in humans [259,260]. Safety evaluation studies on carrageenan suggest that sub-chronic or chronic feeding of this food-grade polysaccharide did not induce any toxic effects [261]. Moreover, dietary supplementation of carrageenan was not associated with carcinogenicity, genotoxicity, or reproductive defects [261]. Another study reported that no toxicological response was induced when iota-carrageenan was administered through the intranasal route [262]. Several studies also investigated toxicity of fucoxanthin and suggested that this carotenoid was safe and caused no visible toxicity in experimental subjects [263,264,265]. The toxicity profiles of some other marine metabolites have recently been reviewed [25]. As sufficient toxicological profiles of other potentially bioactive metabolites are lacking, they should be investigated with appropriate experimental models.

## 8. Conclusions and Future Perspectives

**Figure 5:**
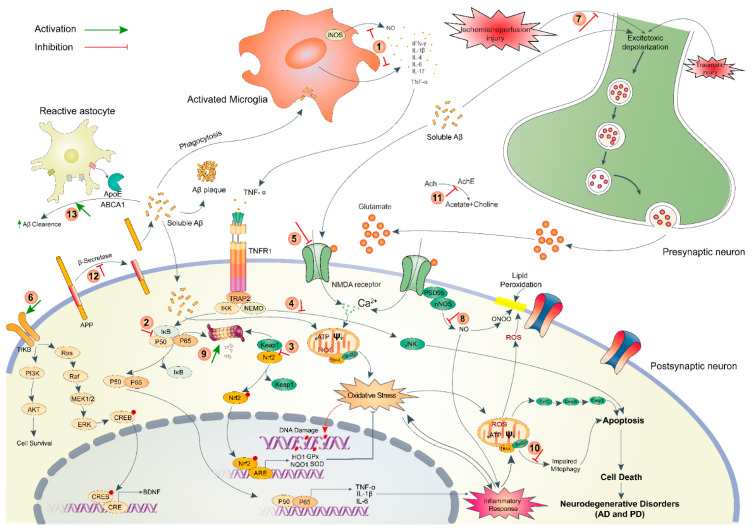
A scheme highlighting the pathophysiology of neurodegenerative disorders and post-ischemic consequences along with indicating the underlying mechanism of neuroprotective action of algal compounds. The numeric symbols indicate the points of pharmacological action that include (1) inhibition of cytokine secretion from activated microglia by fucoxanthin, fucosterol, fucoidan, dieckol, phlorofucofuroeckol and bieckol, κ-carrageenan, floridoside and seleno-polymannarate, (2) attenuation of inflammatory response via inhibition of NF-κB pathway by eckol, dieckol and 8,8-bieckol, (3) priming of antioxidant defense system via activation of Nrf2/ARE pathway (blocking interaction between Nrf2 and Keap1) by fucoxanthin, fucoidan and zonarol, (4) Reduction of apoptosis via inhibiting pro-apoptotic JNK/Erk pathway by dimethylsulfoniopropionate and κ-carrageenan-derived pentasaccharide, (5) Inhibition of glutamate-induced Ca2+ influx via blocking extrasynaptic GluN2B by fucoidan and tramiprosate, (6) Activation of BDNF-dependent pro-survival pathway via inducing PI3K/Akt or TrkB/ERK signaling by fucoxanthin and fucosterol, (7) Attenuation of I/R-injury via preventing excitotoxic depolarization by C-phycocyanin, (8) Inhibition of nNOS sequestration by tramiprosate, (9) proteasomal degradation by fucoidan, (10) Induction of autophagy/mitophagy by fucoxanthin, (11) anticholinesterase activity by fucoidan, fucoxanthin, dieckol and phlorofucofuroeckol, (12) anti-amyloidogenesis via blocking β-secretase activity by fucoxanthin, fucosterol and glycoprotein, and (13) Aβ-clearance via enhancing the transcription of ApoE and ABC transporters genes by fucosterol, saringasterol, and alginate-derived oligosaccharide. NF-κB/p50-pp65, nuclear factor kappa-light-chain-enhancer of activated B cells; Nrf2, nuclear factor erythroid 2-related factor 2; ARE, antioxidant response element; IkB, inhibitor of NF-κB; Keap1, Kelch-like ECH-associated protein 1; JNK, c-Jun N-terminal kinases; GluN2B, N-methyl D-aspartate receptor subtype 2B; PI3K, phosphoinositide 3-kinases; Akt, protein kinase B; MEK1/2, mitogen-activated protein kinase kinase; ERK, extracellular signal-regulated kinases; TrkB, tropomyosin receptor kinase B; CREB, cAMP-response element binding protein; CRE, cAMP response elements; BDNF, Brain-derived neurotrophic factor; AChE, acetylcholinesterase; Ach, acetylcholine; ABCA1, ATP-binding cassette transporter A1; nNOS, neuronal nitric oxide synthase; ROS, reactive oxygen species; ψ, mitochondrial membrane potential.

The current review highlights several neuropharmacological attributes, such as antioxidant, anti-inflammatory, anti-cholinesterase, anti-amyloidogenic, antiaging, protein clearance, cholesterol homeostasis, and neuritogenic capacity of algae-derived metabolites that underlie their neuroprotective functions against a wide range of neurotoxic stimuli (Figure 5). The neuroprotective effects of marine algae and their metabolites do not necessarily depend on a single attribute, rather on the synergism of multiple of these pharmacological properties. As neurodegenerative disorders involve complex pathogenic mechanisms, they could be better managed with a single compound targeting two or more of the pathogenic mechanisms or multiple compounds with the complementary mechanism of action. In this context, algal compounds, such as fucoxanthin, fucosterol, and fucoidan that are known to target multiple pathogenic mechanisms could be potential candidates for future drug development. In addition, several metabolites, including laminarin, porphyran, saringasterol, α-bisabolol, and phlorotannins that exhibited encouraging neuroprotective roles, also deserve further attention.

Although neuroactive compounds were isolated from a range of algae, seaweed species under Phaeophyceae yield the highest number of compounds. However, species from other groups, for example, *Gelidium amansii* under Rhodophyceae that exhibited significant neuromodulatory effects, also could offer some promising metabolites. Moreover, a large number of species remain unexplored. While degenerating brains experience disruption of synaptic connectivity, compounds with neuritogenic capacity may potentially enhance the regeneration of damaged processes. Therefore, compounds, both neuroprotective and neurotrophic, are equally important. However, in contrast to neuroprotective compounds that potentially support neuronal survival, a few compounds showing neurite outgrowth potential have been discovered in marine algae. Compounds, including those that have already shown neuroprotective ability as well as those that have not yet been explored, therefore, need to be screened for their ability to promote neurite extension.

Despite a sizable collection of algae-based natural products with distinct neuroprotective functions, only sodium oligomannate has emerged as a successful drug for AD. This review, therefore, calls for intensive research on other potential compounds to translate the preclinical findings into clinical models. In addition, the factors that are responsible for the failure of a clinical trial need to be carefully reviewed. For example, the bioavailability of a candidate drug in the brain, including its ability to cross BBB, remains one of the barriers to therapeutic success. If the ADME (absorption, distribution, metabolism, and excretion) properties of a preclinically effective compound sufficiently guarantee its drug-likeliness, it is highly likely that the compound may succeed in clinical trials. This is why the ongoing strategy requires a rational reformation incorporating modern approaches, such as virtual screening and system biology, to strengthen the algae-based drug development process. The computational study will provide some crucial information on the ADME properties of potential leads and its interaction and binding affinity to molecular targets while system biology knowledge will identify the potential interaction of target molecules and cellular signaling pathways at the systemic level. With the constant discovery of new compounds, all these strategies will accelerate the designing and development of algae-based future drugs.

## Figures and Tables

**Table 1:** Summary on pharmacological effects, occurrence, effective dose, experimental model, cellular effects, potential pharmacological mechanism of algal metabolites.

Pharmacological Effects	Compound (Class)	Algal Source If Any)	Effective Concentration	Experimental Model (In Vivo/In Vitro)	Cellular Effects/Significant Findings	Signaling Pathways Involved	Pharmacological Markers	Reference
Antioxidant activity	Fucoxanthin (carotenoids)	*Sargassum siliquastrum*	50 and 100 μM	H_2_O_2_-induced cell damage in kidney fibroblast cells	Attenuates oxidative stress	n.d.	↓ROS level	[69]
	Fucoxanthin		5, 10, and 50 μM	H_2_O_2_ induced BV2 microglial cells	Antioxidation	Antioxidant pathway	↓ROS ↑SOD and GSH	[36]
	Fucosterol, 3,6,17-trihydroxy-stigmasta-4,7,24(28)-triene and 14,15,18,20-diepoxyturbinarin (sterols)	*Pelvetia siliquosa*	A seven day-dose regimen at 30 mg/kg/day before carbon tetrachloride (CCl4) administration	Rat model	Antioxidation	n.d.	↑SOD, CAT, and GPx	[71]
	Fucosterol	*Eisenia bicyclis,* brown alga	25, 50, 100, 200, and 400 μM	RAW 264.7 murine macrophages (t-BHP stimulated)	Protects against oxidative stress	n.d.	↓ROS generation	[72]
	Fucosterol	*Ecklonia stolonifera and Eisenia bicyclis;* Brown algae	25, 50, and 100 μM	tert-Butyl hydroperoxide- and tacrine-induced HepG2cell injury model	Antioxidation	n.d.	↓ROS generation ↑GSH level	[73]
	Fucosterol	*Sargassum* *Binderi;* brown alga	3.125, 6.25, 12.5, 25, 50, and 100 μg /mL	Particulate matter-induced injury and inflammation in A549 human lung epithelial cells	Attenuates oxidative stress		↓ROS level ↑SOD, CAT, and HO-1 in the cytosol, and NRF2 in the nucleus	[74].
	Glycoprotein	*U. pinnatifida*	SOD activity and Xox activity at a concentration of 5 mg/mL and 1 mg/mL, respectively	In vitro enzyme assay			↑SOD and↓Xox	[75]
	Sulfated oligosaccharides	*Ulva lactuca* and *Enteromorpha prolifera;* green algae	150 mg/kg·day	Aging model (male senescence-accelerated prone (SAMP8) and male senescence resistant (SAMR1) mice)	Antioxidantion	n.d.	↑GSH, SOD, CAT, telomerase levels, ↑Total antioxidant capacity, ↓MDA and AGEPs	[96]
Anti-inflammatory activity	Fucoxanthin		5, 10, and 50 μM	Aβ_42_-induced BV2 microglia cells	Anti-inflammation	MAPK pathway	↓iNOS, COX-2 ↓TNF-α, IL-6, IL-1β, PGE_2_ ↓JNK, ERK, and p38 MAPK phosphorylation	[36]
	Fucoxanthin	*-*		LPS-activated BV-2 microglia	Anti-inflammation and antioxidation	Akt/NF-κB and MAPKs/AP-1 pathways; PKA/CREB pathway	↓iNOS, COX-2, ↓TNF-α, IL-6, PGE_2_, NO, ROS ↓IL-6, TNF-α, iNOS, and COX-2 mRNA expression ↓Akt, NF-κB, ERK, p38 MAPK and AP-1 phosphorylation ↑Nrf2, HO-1 ↑PKA, CREB ↑BDNF	[70]
	Fucosterol	*E. bicyclis;* brown alga	5–20 μM for NO	RAW 264.7 murine macrophages (t-BHP 200 μM, LPS-1μM stimulated)	↓Inflammatory response	↓NF-κB pathway	↓NO production ↓iNOS and COX-2	[72]
	Fucosterol	*U. pinnatifida*	10, 25, or 50 μM	LPS-induced RAW 264.7 macrophages and THP-1 human monocyte cell line	↓Inflammatory response	↓NF-κB pathway	↓iNOS, TNF-α, and IL-6 ↓DNA binding ↓phosphorylation of NF-κB, MKK3/6 and MK2	[83]
	Fucosterol	*Hizikia fusiformis*	1–10 μM	CoCl_2_ induced hypoxia in keratinocytes	↓Inflammatory response	n.d.	↓IL-6, IL-1β and TNF-α ↓pPI3K and pAkt and HIF1-α accumulation	[82]
	Fucosterol	*Panida. australis*	0.004,0.2, and 10 μM	LPS or Aβ-induced BV2 (microglial) cells	Protects against LPS or Aβ-mediated neuroinflammation	n.d.	↓IL-6, IL-1β, TNF-α, NO, and PGE2	[85]
	Fucosterol	*S. Binderi;* brown alga	3.125, 6.25, 12.5, 25, 50, 100 μg/mL	Particulate matter-induced injury and inflammation in A549 human lung epithelial cells	↓Inflammatory response	n.d.	↓COX-2, PGE2, TNF-α and IL-6	[74]
	Dieckol (phlorotannin)	*E. cava*	50–300 µg/mL	LPS-stimulated murine BV2 microglia	Anti-inflammation and antioxidation	p-38 MAPK/ NF-κB pathway	↓NO and PGE_2_; ↓iNOS and COX-2; ↓IL-1β and TNF-α; ↓ROS	[86]
	Phloroglucinol, eckol, dieckol, 7-phloroeckol, phlorofucofuroeckol A and dioxinodehydroeckol (phlorotannin)	*E. bicyclis;* brown alga	5–20 μM for NO	LPS-stimulated RAW 264.7 murine macrophages	↓Inflammatory response	↓NF-κB pathway	↓NO production	[72]
	Phlorofucofuroeckol A	*E. stolonifera*	20 μM	LPS-activated BV2 and primary microglial cells	Anti-inflammation	NF-κB, JNKs, p38 MAPK, and Akt pathways	↓NO and PGE_2_; ↓iNOS and COX-2; ↓IL-1β, IL-6 and TNF-α; ↓NF-κB activation and IκB-α degradation ↓JNK, p38, and Akt	[87]
	Phlorofucofuroeckol B (phlorotannin)	*E. stolonifera*	10–40 µM	LPS-stimulated murine BV2 microglia	Anti-inflammation	IκB-α/NF-κB and Akt/ERK/JNK pathways	↓TNF-α, IL-1β and IL-6; ↓COX-2 and iNOS ↓NF-κB activation and IκB-α degradation ↓Akt, ERK, and JNK phosphorylation	[88]
	8,8’-bieckol (phlorotannin)	*E. cava*		LPS-stimulated primary macrophages and RAW 264.7 macrophages & LPS-induced septic mice	Anti-inflammation; Protects mice from endotoxin shock	NF-κB pathway	↓NO and PGE_2_; ↓iNOS mRNA and protein expression; ↓IL-6; ↓Transactivation of NF-κB and nuclear translocation of the NF-κB p65 subunit ↓ROS	[90]
	6,6′-bieckol (phlorotannin)	*E.* *stolonifera*		LPS-stimulated BV2 and murine primary microglial cells	Anti-inflammation	IκB-α/NF-κB and JNK/p38 MAPK/Akt pathways	↓COX-2 and iNOS; ↓NO and PGE_2_, ↓IL-6 ↓Transactivation of NF-κB and nuclear translocation of the NF-κB p65 subunit ↓Akt, JNK and p38 MAPK phosphorylation	[89]
	Fucoidan (sulfated polysaccharide)	Brown seaweed	25, 50, and 100 µg/mL	LPS-stimulated murine BV2 microglia	Anti-inflammation	NF-κB and JNK/p38 MAPK/Akt pathways	↓NO and PGE_2_; ↓COX-2, iNOS and MCP-1; ↓TNF-α and IL-1β; ↓NF-κB activation; ↓Akt, ERK, p38 MAPK and JNK phosphorylation	[92]
	Fucoidan	-	125 µg/mL	LPS-activated primary microglia	Anti-inflammation	n.d.	↓TNF-α and ROS	[93]
	κ-carrageenan oligosaccharides and desulfated derivatives	Red algae		LPS-activated microglia	Anti-inflammation	n.d.	↓TNF-α	[94]
	Sulfated oligosaccharides	*U. lactuca* and *E. prolifera;* green algae	150 mg/kg·day	Aging model (male senescence-accelerated prone (SAMP8) and male senescence resistant (SAMR1) mice)	↓Inflammatory response	n.d.	↓IFN-γ, TNF-α, and IL-6	[96]
	Alginate-derived oligosaccharide	Brown algae	50–500 µg/mL	LPS/Aβ-stimulated BV2 microglia	Anti-inflammation	TLR4/NF-κB signaling pathway	↓NO and PGE_2_; ↓COX-2 and iNOS; ↓TNF-α, IL-6 and IL-12; ↓TLR4; ↑NF-κB/p65 subunit translocation	[97]
	Seleno-polymannuronate	Brown algae	0.8 mg/mL	LPS-activated primary microglia and astrocytes; mouse model of acute inflammation	Anti-inflammation	NF-κB and MAPK signaling	↓NO and PGE_2_; ↓COX-2 and iNOS; ↓TNF-α, IL-1β and IL-6; ↑IκB-α, p65, p38, ERK and JNK phosphorylation	[98]
	Sargachromenol (plastoquinone)	*Sargassum micracanthum*	30.2 μM (IC_50_)	LPS-stimulated RAW 264.7 macrophages	Anti-inflammation	NF-κB signaling	↓NO and PGE_2_; ↓COX-2 and iNOS; ↑IκB-α	[99]
	Sargaquinoic acid (plastoquinone)	*Sargassum siliquastrum*		LPS-stimulated RAW 264.7 macrophages	Anti-inflammation	NF-κB signaling	↓NO; ↓iNOS; ↑IκB-α; ↓nuclear translocation of NF-κB; ↓JNK1/2 MAPK	[100]
	Floridoside (glycerol glycosides)	*Laurencia undulate;* red alga	50 μM	LPS-stimulated murine BV2 microglia	Anti-inflammation	MAPK Signaling	↓NO, ROS; ↓iNOS and COX-2; ↓p38 MAPK and ERK phosphorylation	[101]
	Glycoprotein	*U. pinnatifida*	COX-1 and COX-2 inhibition with IC_50_ values of 53.03 ± 1.03 μg/mL and 193.35 ± 3.08 μg/mL, respectively	LPS-stimulated RAW 264.7 macrophages	Anti-inflammation	n.d.	↓COX-1 and COX-2 ↓NO	[75]
	Caulerpin (bisindole alkaloid)	*Caulerpa racemosa*	100 µM/kg body wt	Capsaicin-induced ear edema and carrageenan-induced peritonitis	Inhibition of nociception	n.d.	n.d.	[130]
	Caulerpenyne (sesquiterpene)	*C. prolifera* and *C. racemosa*	5.1 μM	Lipoxygenase (LOX) enzyme activity assay	Inhibitory activity against LOX	-	Un-competitive type of inhibition	[131]
	Aquamin (multi-mineral complex)	*Lithothamnion corallioides*; red alga		LPS-stimulated, glial-enriched primary cultures of rat cortex	Anti-inflammation	n.d.	↓TNF-α and IL-1β	[132]
Anticholinesterase activity	Fucosterol and 24-hydroperoxy 24-vinylcholesterol	*E. stolonifera*	IC_50_ values of 421.72 ± 1.43, 176.46 ± 2.51 µM, respectively	In vitro enzymatic assay	↓BChE activity	-	Selective inhibition of BChE	[114]
	Fucosterol	*Panida australis*	inhibition against AChE (10.99–20.71%) and BChE (4.53–17.53%) with concentrations ≤ 56 μM,	In vitro enzymatic assay	↓AChE and BChE activities	-	Nonselective cholinesterase inhibition	[85]
	Fucosterol	*Sargassum horridum*	-	In vitro enzymatic assay	↓AChE activity	-	Non-competitive inhibition	[115]
	Fucoxanthin	*-*	IC_50_ value 1.97 mM	In vitro BChE activity assay	↓BChE activity		Mixed inhibition type	[116].
	Fucoxanthin	Brown seaweed	IC50 value of 81.2 μM	In vitro AChE activity assay; Molecular docking analysis	↓AChE activity	Fucoxanthin likely interacts with the peripheral anionic site within AChE	Non-competitive manner	[117]
	α-Bisabolol	*Padina gymnospora*	IC50 value < 10 μg/mL	In vitro enzymatic assay	↓AChE and BChE activity	-	-	[118]
	Glycoprotein	*U. pinnatifida*	AChE and BChE inhibitory activities with IC_50_ values of 63.56 ± 1.86 and 99.03 ± 4.64, respectively	In vitro enzymatic assay	↓AChE and BChE activity	-	-	[75]
	Phloroglucinol, dibenzo [1,4] dioxine-2,4,7,9-tetraol and eckol	*Ecklonia maxima;* Brown alga	IC50 value: 76.70 to 579.32 μM	In vitro AChE activity assay	↓AChE activity	-	-	[119]
	Dieckol and phlorofucofuroeckol	*E. cava*		Ethanol-intoxicated memory impairment in mice	↓AChE activity	n.d.	↑Acetylcholine	[120]
	Sargaquinoic acid and sargachromenol (plastoquinones)	*Sargassum sagamianum*	IC_50_ value for anti-AChE: 23.2 and 32.7 μM, respectively; IC_50_ value for anti-BChE of sargaquinoic acid 26 nm	In vitro ChE activity assay	Sargaquinoic acid shows potent inhibitory activity against BuChE and moderate inhibitory activity against AChE	-.	-	[121]
	(5E,10Z)-6,10,14-trimethylpentadeca-5,10-dien-2,12-dione and (5*E*,9*E*,13*E*)-6,10,14-trimethylpentadeca-5,9,13-trien-2,12-dione (Sesquiterpenes)	*S. sagamianum*	IC_50_ values of 65.0 and 48.0, and 34.0 and 23.0 μM, respectively	In vitro ChE activity assay	Moderate inhibitory activity against AChE and BuChE	-	-	[133]
Anti-amyloidogenic and aggregation inhibition activity	Fucoxanthin	*E. stolonifera and U. pinnatifida*			↓β-secretase activity; Binding energy (-7.0 kcal/mol)	-	mixed-type inhibition	[134]
	Fucoxanthin	*-*	0.1–30 μM		Suppresses the formation of Aβ1-42 fibrils and Aβ1–42 oligomers, and inhibits Aβ aggregation	-	-	[135]
	Fucoxanthin	*-*	2 μM	ThT assay	Inhibits Aβ1-42 fibril and aggregate formation	-	-	[136]
	Fucosterol	*E. stolonifera and U. pinnatifida*	10–100 μM (IC_50_ value of 64.12 ± 1.0 μM)	In vitro enzyme assay; In silico analysis	↓β-secretase activity; Binding energy (−10.1 kcal/mol)	-	Noncompetitive inhibition	[134]
	α-Bisabolol	*Padina gymnospora*	5 μg/mL	Thioflavin T (ThT), Confocal laser scanning microscopy (CLSM) analysis, Transmission electron microscopy (TEM), Fourier transform infrared (FTIR) spectroscopic analysis and molecular dynamics simulation	Prevents oligomers formation as well as disaggregates the matured fibrils	-	-	[137]
	Glycoprotein	*U. pinnatifida*	IC_50_ values of 73.35 ± 2.54 μg/mL	*In vitro* enzymatic assay	↓BACE1 activity	-	-	[75]
Cholesterol homeostasis and Aβ clearance activity	Fucosterol	-	100 and 200 μM (HEK293 cell cultures); 100 or 200 μM (macrophages and HepG2, H4IIE, and Caco_2_ cells)	HEK293 cell cultures (Reporter system); THP-1-derived macrophages; Caco-2 cells HepG2 cells	Reverses cholesterol transport. No accumulation of triglyceride in HepG2	n.d.	Dual-LXR agonist (LXR-α and LXR-β) ↑ABCA1, ABCG1, and ApoE; ↑Intestinal NPC1L1 and ABCA1; ↑Insig-2a, that delays nuclear translocation of SREBP-1c	[138]
	Saringosterol	*Sargassum fusiforme*	30 μM	Luciferase reporter assay system; HEK293T, THP-1 monocytes, HepG2, RAW264.7, THP-1 macrophages and Caco-2 cells	n.d.	n.d.	Selective LXRβ agonist; ↑ABCA1, ABCG1, and SREBP-1c	[139]
	Alginate-derived oligosaccharide	Marine brown algae		BV2 microglial cells	Microglial phagocytosis of Aβ	Toll-like receptor signaling	↑TLR4	[97].
*Monoamine oxidase inhibition and affinity to dopaminergic receptors*	Phlorofucofuroeckol-A and dieckol (phlorotannin)	*-*		In vitro enzyme assay and functional assay for GPCR screening; Docking analysis	↓*h*MAO activity; D_3_R and D_4_R stimulation	-	-	[140].
Antiaging	Sulfated oligosaccharides	*U. lactuca* and *E. prolifera;* green algae	150 mg/kg/day	Aging model (male senescence-accelerated prone (SAMP8) and male senescence resistant (SAMR1) mice)	Antioxidant and anti-inflammation	n.d.	↑GSH, SOD, CAT, telomerase levels, ↑Total antioxidant capacity, ↓MDA and AGEPs ↓IFN-γ, TNF-α, and IL-6 ↑BDNF and ChAT; ↑Sirt1, ↑p53 and FOXO1	[96]
	Fucosterol	*Hizikia fusiformis*	50 µg/mL	Culture model of *C. elegans*	Extends lifespan	↑Antioxidant mechanism	n.d.	[141]

n.d.: not defined; -: information not available.

**Table 2:** Neurotrophic activity of algal phytochemicals in vitro.

Compound	Algal Origin (If Any)	Dosage	Experimental Model (In Vivo/In Vitro)	Cellular Effects/Significant Findings	Pharmacological Markers	References
Sargachromenol	*Sargassum macrocarpum* (Brown alga, Japan)	ED_50_ 9 μM (with 10 ng/mL NGF)	PC12D cells	NGF-dependent neurite outgrowth and survival	↑PKA and MAPK1/2 ↑PI3K	[145]
Sargaquinoic acid	*S. macrocarpum* (Brown alga, Japan)	3 µg/mL (with 10 ng/mL NGF)		Cell differentiation	Protein Kinase A and MAP Kinases-Mediated Signaling Pathways	[146]
Vitamin B12 (chlorophyll-related analog to pheophytin)	*Sargassum fulvellum* (Brown alga, Japan)		PC12 cells	Cell differentiation	MAPK signal transduction pathway	[148]
Pheophytin A	*S. fulvellum* (Brown alga, Japan)	3.9 µg/mL	PC12 cells	NGF-independent neurite outgrowth	↑PKA and MAPK1/2 ↑PI3K	[147]
Dimethylsulfoniopropionate	-	7.4 mM	Neuronal N2a and glial OLN-93 cells	Process outgrowth; microtubule reorganization and bundling	↑α-tubulin acetylation	[149]
Fucoxanthin	-	0.1–2 μM	PC-12 cells	NGF-independent neurite outgrowth	n.d.	[136]

n.d.: not defined; -: information not available.

**Table 3:** Neuroprotective activity of algal compounds in vitro and in vivo.

Compound (Class)	Algal Origin (If Any)	Effective Concentration	Experimental Model (In Vivo/In Vitro)	Cellular Effects/Significant Findings	Signaling Pathways Involved	Pharmacological Markers	References
**In Vitro Experimental Models**							
Zonarol (*p*-hydroquinone sesquiterpene)	*Dictyopteris undulate* (Brown alga, Japan)	ED_50_ 0.22 µM (therapeutic index, defined as the ratio of ED_50_ to LD_50,_ is 14.2-fold)	HT22 hippocampal neuronal cells (glutamate-induced oxidative stress) & Cerebrocortical neurons (glutamate or rotenone-induced oxidative stress)	Neuronal survival against oxidative stress	Nrf2/ARE pathway	↑NQO-1, HO-1, and PRDX4	[170]
Fucoxanthin	*Undaria pinnatifida*	0.15–1.5 µmol/L	Hypoxia/reoxygenation-induced neuronal injury	Neuronal survival against oxidative stress	n.d.	n.d.	[166]
Fucoxanthin	-	20 μM	*In Vitro* model of TBI (primary culture of mouse cortical neurons scratched manually)	Neuronal survival against secondary injury (oxidative stress)	Nrf2-ARE and Nrf2-autophagy pathways	↓ROS ↑Beclin-1 (Atg6), LC3 (Atg8) and↓p62 ↓Cleaved caspase-3 ↑Nrf2 nuclear translocation ↑HO-1 and NQO-1	[169]
Fucoxanthin	-	3 μM	β-Amyloid oligomer-induced neurotoxicity in SH-SY5Y Cells	Neuronal survival against oxidative stress	PI3K/Akt and ERK Pathways	↓ROS ↑pSer473-Akt and pSer9-GSK3*β* ↓pERK	[164]
Fucoxanthin	-	1-3 μM	H_2_O_2_-induced toxicity in SH-SY5Y Cells and primary cerebellar granule neurons	Neuronal survival against oxidative stress	PI3K/Akt and ERK Pathways	↓ROS ↑pSer473-Akt and pSer9-GSK3*β* ↓pERK	[165]
Fucoxanthin	-	0.3 μM	Fucoxanthin-modified Aβ_1–42_ oligomers-induced neurotoxicity in SH-SY5Y Cells	Neuronal survival	n.d.	n.d.	[135]
Fucoxanthin	-	5 μM, 10 μM, and 20 μM	Oxygen-glucose deprivation and reoxygenation (OGD/R) model of cultured neurons	Neuronal survival against oxidative stress	Nrf2/HO-1 signaling	↑Nrf2 nuclear translocation ↑HO-1	[168]
Fucoxanthin	*Undaria pinnatifida*	0.075 μg/mL	H/R-induced excitotoxicity in primary hippocampal neurons	Neuronal survival against oxidative stress	n.d.	n.d.	[167]
Fucoxanthin	-	<2 μM (against Aβ1-42-mediated toxicity) 0.5–2 μM(H_2_O_2_-induced cytotoxicity)	Aβ1-42-mediated toxicity in PC12 cells H_2_O_2_-induced cytotoxicity	Cell survival	n.d.	n.d.	[136]
α-Bisabolol	*Padina gymnospora*	5 μg/mL	Aβ25-35-induced neurotoxicity in PC-12 cells	Antiapoptosis	n.d.	n.d.	[137]
α-Bisabolol	*Padina gymnospora*	5 and 10 μg/mL	Aβ25-35-induced neurotoxicity in Neuro2a cells and transgenic *C. elegans*	Antioxidation Antiapoptosis; Protection against Aβ induced proteotoxicity	Aβ mediated pathway	↓ROS, NOS ↓Bax and caspase-3 ↓ace-1, hsp-4 and Aβ	[171]
Fucosterol	***Ecklonia stolonifera***	1–10 µM at 24 h before sAβ1-42 exposure (effective fucosterol conc. 5–10 µM)	sAβ1-42 (10 µM)-induced ER stress model of primary neurons	Attenuates Aβ1-42-induced neurotoxicity	n.d.	↑TrkB-mediated ERK1/2 signaling ↓GRP78 expression ↑BDNF expression	[172]
Fucosterol	-	0.0032 to 20 μM	Aβ-induced cytotoxicity in SH-SY5Y cells	Reduces apoptosis in Aβ-induced SH-SY5Y cells	n.d.	↑Ngb mRNA ↓APP mRNA and intracellular Aβ levels	[173]
Eckol, dieckol and 8,8′-bieckol	*Ecklonia cava*	1–50 µM	Aβ25-35-stimulated PC12 cells	Antioxidation, anti-inflammation, anti-apoptotic properties	NF-κB pathway	↓COX-2, iNOS; ↓TNF-α, IL-1β and PGE_2_ production; ↓p38, ERK and JNK	[96]
Phloroglucinol, eckol, triphloroethol A, eckstolonol, and dieckol	*Ecklonia cava*	50 μM	H_2_O_2_-induced oxidative stress in murine hippocampal HT22 cells	↓Lipid peroxidation; ↓apoptosis	n.d.	↓ROS ↓Ca^2+^ release	[178]
Diphlorethohydroxycarmalol	*Ishige okamurae*	50 μM	H_2_O_2_-induced oxidative stress in murine hippocampal HT22 cells	Antioxidation; ↓Lipid peroxidation; ↓Apoptosis	n.d.	↓Bax ↑Bcl-xL ↓Poly (ADP-ribose) polymerase-1 (PARP) cleavage ↓ROS ↓Ca^2+^ release	[179]
Phloroglucinol, dioxinodehydroeckol, eckol, phlorofucofuroeckol A, dieckol, and 7-phloroeckol	*Eisenia bicyclis*	2.5, 5, 10 and 20 µg/mL	Aβ peptide-induced toxicity in PC12 cells	Antioxidation	n.d.	↓ROS ↓Ca^2+^ release	[175]
Phlorofucofuroeckol	Brown algae	10 µm	Glutamate-induced cytotoxicity in PC12	Antioxidation	n.d.	↓Caspase-3, -8, and PARP	[180]
Eckmaxol (phlorotannin)	*Ecklonia maxima*	20 µm	β-amyloid oligomer -induced neuronal apoptosis in SH-SY5Y cells	↓Apoptosis	GSK-3β and ERK pathways	↑pGSK-3β ↓pERK ↑HO-1	[181]
Fucoidan	-	0.1–1.0 µm	Aβ_1−42_-induced neurotoxicity in rat cholinergic basal forebrain neurons	Restores Aβ-induced reduction in whole-cell currents	n.d.	↑pPKC ↓ROS ↓caspases 9 and 3	[182]
Fucoidan (sulfated polysaccharide)	-	0.1 and 1.0 mg/mL	MPP(+)-induced injury in MN9D cells	Antioxidation; Protects cellular injury	n.d.	n.d.	[183]
Fucoidan (sulfated polysaccharide)	-	60 and 30 μg/mL	H_2_O_2_-induced apoptosis in PC12 cells	↑Cell viability; antioxidation	PI3K/Akt signaling	↓ROS; ↑SOD and GPx activities; ↓MDA; ↑Bcl-2/Bax ratio; ↓caspase-3; ↑p-Akt	[184]
Fucoidan (sulfated polysaccharide)	-	100, 200, 400 μg/mL	Aβ25–35 and d-Gal-induced neurotoxicity in PC12 cells	↓Apoptosis	Caspase-dependent apoptosis pathway	↓Cytochrome c release; ↓Caspase activation; ↑Livin and XIAP; ↑SOD ↑GSH	[185]
Fucoidan (sulfated polysaccharide)	-	100 μM	MPP(+)-induced injury in dopaminergic precursor cell line(MN9D) cells	↓Apoptosis; Antioxidation;	CatD-Bax signaling axis	↓LC3-II and CatD; ↓Bax; ↑SOD ↑GSH	[186]
Fucoidan (sulfated polysaccharide)	Fucus vesiculosus Linn., brown alga	0.5 mg/mL or 1.5 mg/mL	NMDA-induced Ca^2+^ responses in culture rat neurons	Suppresses the intracellular Ca^2+^ responses by selectively inhibiting NMDA receptors in cortical neurons and l-type Ca^2+^ channels in hippocampal neurons.	n.d.	↓GluNR1 mRNA and l-type Ca^2+^ channels, PR1/PR2	[187]
Oligo-porphyran	Synthesized from porphyran (isolated from *Pyropia yezoensis*) through acidolysis reaction	200 μg/mL	6-OHDA-induced cytotoxicity in PC12 cells	↓Apoptosis; Antioxidation; Anti-inflammation	PI3K/ Akt/PKC pathway	↓ROS; ↑MMP ↑SOD and GSH; ↑Bcl-2/Bax ratio; ↓caspase-3 and -9 ↑p-Akt, p-PI3K, PKC ↑DAT and TH ↓TNF-α, IL-1β, and IL-6	[188]
Acidic oligosaccharide sugar chain	*Echlonia kurome* Okam	50, 75, 100 μg/mL	Inflammatory responses and cytotoxicity in SH-SY5Y cell line induced by Aβ-stimulated astrocytes conditioned medium	Oxidative stress	n.d.	↓TNF-α and IL-6; ↓Ca^2+^ influx	[189]
Racemosins A (bisindole alkaloid)	*Caulerpa racemosa,* green alga	10μM	Aβ25–35-induced SH-SY5Y cell damage	↑Cell viability; ↓apoptosis	n.d.		[190]
Tramiprosate (small aminosulphonate compound)	Red marine algae	50 mg/kg	OGD- or NMDA-induced injury in NGF-differentiated PC12 cells and primary cortical neurons	Protects against neuronal injury	n.d.		[191]
Dimethylsulfoniopropionate	-	1 mg/mL	Tropodithietic acid -induced cytotoxicity in OLN-93 and N2a cells	Protects against neurotoxicity; Attenuates stress responses and mitochondrial damage	n.d.	↓ERK1/2 activation and HSP32 induction	[149]
κ-Carrageenan-derived pentasaccharide	marine red algae	25, 50, or 100 µM	Aβ25-35-induced neurotoxicity in SH-SY5Y cells	↑Cell viability; ↓Apoptosis	JNK signaling pathway	↓Cleaved caspase 3 ↓p-JNK	[192]
**In vivo experimental models**							
Fucoidan (sulfated polysaccharide)	-	25 mg/kg	MPTP-induced animal model of Parkinsonism in C57/BL mice in vivo	↓Behavioral deficits; ↓TH-positive neuronal loss	n.d.	↑Dopamine, DOPAC and HVA; ↑Tyrosine hydroxylase; ↑GSH; ↑SOD, GPx, and catalase activity and total antioxidant capacity;	[183]
Fucoidan (sulfated polysaccharide)	-	7.5 and 15 mg/kg body wt (intranigral injection)	LPS-induced neurotoxicity in rat	Ameliorates behavioral deficits, prevents the loss of dopaminergic neurons and inhibits the deleterious activation of microglia in the substantia nigra pars compacta	n.d.	↓CD11b	[93]
Fucoidan (sulfated polysaccharide)	-	50, 100, 200 mg kg^−1^	Aβ1-40-induced learning and memory impairment in rats	Ameliorates learning and memory impairment; ↓oxidative stress; ↓apoptosis	Antioxidation	↑ChAT, SOD and GPx activity; ↑Ach; ↓AchE activity; ↓MDA; ↑Bcl-2/Bax ratio; ↓caspase-3 activity	[193]
Fucoidan (sulfated polysaccharide)	-	100 and 200 mg/kg on day 2–6, 50 mg/kg on day 4–6	d-Gal-induced cognitive dysfunction in mice	↓Apoptosis; ameliorate the learning and memory impairment	Caspase-dependent apoptosis pathway	↑Ach level and ChAT activity; ↓AChE activity; ↑SOD; ↑GSH	[185]
Fucoidan (sulfated polysaccharide)	-	100–500 ng/mL	Transgenic *C. elegans* AD model	Alleviates the paralyzed phenotype; ↓Aβ deposits	n.d.	↑Proteasomal activity (proteolysis); ↓ROS	[194]
Fucoidan-rich substances	*E. cava*	Polyphenol/fucoidan extract and mixture (4:6)	Trimethyltin-induced cognitive dysfunction model	Ameliorates learning and memory impairment	n.d.	↓ROS; ↑MMP; ↓BAX and cytochrome C release; ↓Amyloid β production; ↓Tau hyperphosphorylation	[195]
Fucoidan	-	50 mg/kg	Transient global cerebral ischemia (tGCI) model of gerbils	↓Oxidative stress and glial activation	n.d.	↑SOD1 and SOD2	[196]
Laminarin (polysaccharide)	-	50 or 100 mg/kg (i.p) for seven days before IR (5-min transient ischemia) surgery	Forebrain I/R injury in young gerbils (6 months)	↓Reactive gliosis (M1 microglia) and neuroinflammation	n.d.	↓IL-2	[197]
Laminarin (polysaccharide)	Brown algae	50 mg/kg/day (i.p) for seven days before IR (5-min transient ischemia) surgery	Forebrain I/R injury in aged gerbils (22–24 months)	↓Oxidative stress and neuroinflammation	n.d.	↓Superoxide anions and 4-hydroxy-2-nonenal (HNE) ↓IL-1β and TNF-α ↑SOD1 and SOD2 ↑IL-4 and IL-13	[198]
Oligo-porphyran	Synthesized From porphyran (isolated from *Pyropia yezoensis*) through acidolysis reaction	25 and 50 mg/kg	6-OHDA-induced Parkinsonian mice model	↓Apoptosis; Ameliorates behavioral deficits	PI3K/Akt/Bcl-2 pathway	↑DAT and TH; ↓caspase-3 and -9 ↑DA, NE, 5-HT, DOPAC ↑p-ERK1/2, DRD2 ↑p-Akt, p-PI3K, GSK-3β ↑Bcl-2/Bax ratio; ↓PARP and cytC ↑p-TrkA and NGF	[199]
Porphyran (polysaccharide)	Degraded polysaccharide from *Pyropia haitanensis*	75, 150, 300 mg/kg	Aβ1-40-induced mice AD model	Improved learning and memory deficits	n.d.	↑ChAT activity; ↓AChE activity; ↑Ach	[200]
Fucoxanthin	Brown seaweed	50, 100, 200 mg/kg	Scopolamine-induced cognitive impairments in mice	Memory enhancement; anticholinesterase	n.d.	↓AChE and choline acetyltransferase ↑BDNF	[117]
Fucoxanthin	-	0.1−30 μM	Aβ oligomer-induced cognitive impairments in mice	Memory enhancement, attenuation of oxidative stress	n.d.	↑BDNF	[135]
Fucoxanthin	-	5 μM, 10 μM, and 20 μM	Middle cerebral artery occlusion (MCAO) rat model (cerebral ischemic/reperfusion (I/R) injury)	Improves the neurologic deficit score and reduces the infarct volume	n.d.	↑SOD activity ↓ROS, MDA ↓cleaved caspase-3 ↑Bcl-2/Bax ratio	[168]
Fucoxanthin		100 mg/kg and 0.05 mmol/L	Traumatic brain injury (TBI) model	Anti-apoptosis, attenuation of oxidative stress, induction of autophagy	Nrf2-ARE and Nrf2-autophagy pathways	↑GPx ↓MDA ↓Cleaved caspase-3, PARP, cytosolic cytochrome c ↑Mitochondrial cytochrome c ↑Beclin-1 (Atg6), LC3 (Atg8) and↓p62 ↑Nrf2 nuclear translocation ↑HO-1 and NQO-1	[169]
Fucosterol	*Ecklonia stolonifera*	1–10 µM	sAβ1-42-induced memory dysfunction in aging rats	Ameliorates Aβ1-42-induced memory impairment	n.d.	↑TrkB-mediated ERK1/2 signaling ↓GRP78 expression ↑BDNF expression	[172]
Dieckol and phlorofucofuroeckol	*Ecklonia cava*	PFF (0.2 and 2 mg/kg) and dieckol (1 and 10 mg/kg)	Ethanol-intoxicated memory-impaired mice	↓AChE activity; reduces the inhibition of latency	n.d.	↑ACh	[120]
C-Phycocyanin		200 mg/kg	Global cerebral ischemia/reperfusion (I/R) injury in gerbils	Reduces the infarct volume and improves the neurologic deficit score; protects neurons, improves the functional outcome (locomotor behavior) and promotes survival	n.d.	↓MDA	[201]
Tramiprosate (small aminosulphonate compound)	Red marine algae	50 mg/kg	Intraluminal filament model of MCAO	Reduces infarct volume	PSD95/nNOS signaling	Disruption of the interaction between PSD95 and nNOS; ↓nNOS translocation	[191]
Sulfated agaran	*Gracilaria cornea*, red alga	60 μg, single intrastriatal injection	Rat 6-hydroxydopamine Parkinson’s disease model	↓Oxidative/ nitrosative stress; restores behavioral deficits and locomotor performance; improves weight	n.d.	↑DA, DOPAC and HVA; ↓5-HT; ↓NO2/NO3 and TBARS; ↑GSH; ↓p65, IL-1β and iNOS; ↑BDNF	[202]

n.d.: not defined; -: information not available.
